# Too young to ask their own questions: Learning and visitor dynamics in a constructivist museum environment

**DOI:** 10.1371/journal.pone.0355543

**Published:** 2026-08-21

**Authors:** Nina Wanča, Jakub Jehlička, Anna Hála

**Affiliations:** 1 Faculty of Arts, Charles University, Prague, Czech Republic; 2 National Museum, Prague, Czech Republic; Universiti Teknologi Malaysia - Main Campus Skudai: Universiti Teknologi Malaysia, MALAYSIA

## Abstract

In 2023, the Children’s Museum, a new permanent exhibition for children, opened at the National Museum in Prague. Primarily designed for children aged 5–10, the exhibition aims to provide an educational alternative to traditional (pre-)school education and classical museums. Through a combination of interactive and traditional presentation methods, the exhibition offers imaginative and interdisciplinary perspectives on various phenomena. It encourages visitors to explore and learn in their own ways. This approach requires a significant interpretative effort from both children and their accompanying adults. To evaluate how the exhibition’s concept resonates with visitors, we observed visitor interactions over a period of 2.5 months, analysing 720 interactions involving more than 2000 visitors. Our analysis revealed that older children (aged 6–10, the primary target group) showed higher levels of interaction and engagement compared to younger children. However, the primary target group constituted only 23% of the child visitors, with 72% being younger children. This indicates that the exhibition is not attracting its intended audience effectively, and the majority of the young visitors may face challenges due to the complexity of the exhibition’s concept. We discuss the implications of these findings and suggest strategies to align the exhibition’s content better with its target audience.

## Introduction

Museums, and especially exhibitions designed specifically for children, are very important for informal learning and for building a lifelong relationship with cultural heritage [1]. In addition, family visits tend to focus on spending quality time together (the social aspect) and, while learning may occur, it is not the primary goal. Families may not have specific learning objectives in mind and their visit may be more spontaneous and self-directed [2]. As a result, child-friendly exhibitions use different types of displays and approaches, e.g., hands-on displays, games, cooperative activities, reduced text, emotional learning, experiential displays, use of technology, as well as special exhibition areas for running, moving or relaxing.

These strategies define many dedicated museum exhibitions for children around the world, which represent different approaches to balancing educational and playful aspects of visitor experience. Importantly, we are not referring here to science centers that primarily focus on interactive technologies and STEM disciplines, but rather to generalist museum exhibitions with a broader focus. Examples of such institutions include *Eureka! The National Children’s Museum* in Halifax (UK), *Explora – Il Museo dei Bambini* in Rome (Italy), *Musée des Enfants* in Ixelles (Belgium), *Tropenmuseum Junior* in Amsterdam (Netherlands), *Boston Children’s Museum* (USA), *Amuzeum* in Casablanca (Morocco), or *Seoul Children’s Museum* (South Korea). As family-oriented children’s museums become increasingly common across diverse regions, this exhibition model has become institutionalized as a socially expected standard.

However, learning is not the most important thing for families. Instead, supporting efforts for making meaning has been at the heart of the design process for the designers of the new Children’s Museum (ChM) at the National Museum in Prague. The main concept used in the exhibition’s design is constructivism, which is currently one of the most influential pedagogical approaches [3]. This study aims to find out what kind of learning takes place in the context of constructivist didactics during a family visit at ChM and what are the qualities of social interaction within the family or for other visitors and museum guides; this, given that constructivism is well linked to collaborative learning.

Constructivist theory suggests that children actively construct their own understanding of the world through hands-on experiences, social interactions and self-directed exploration; thus, building on their existing knowledge and experiences to create new meanings and concepts. This theory, developed by Piaget [4] and Vygotsky [5], emphasises the learner’s role as an active participant in the learning process, rather than a passive recipient of information, and highlights the importance of social and cultural contexts in shaping learning. This means that people’s understanding of the same thing may differ because, during the learning process, each person builds on different previous knowledge and experience. As a result, the teacher takes on more the role of a facilitator who supports the students’ active learning process [6]. Compared to the first formulation of the constructivist approach in the 1970s, the current implementation of the paradigm into practice in Czech schools is not at a high level. Despite numerous curricular reforms in the last more than 20 years [7]. Medová, Bulková, and Čeretková [8] pointed out that the lack of expertise in constructivist practice combined with a lack of experience with one’s own constructivist learning is a limiting factor for future teachers’ implementation of the student-centred approach. The latter stands at the heart of the constructivist concept. Understanding how teachers and schools practice constructivist didactics, and what teachers’ ideas about the concept are, were an important part of formulating our research. We assumed that the understanding would overlap to that of museum professionals, given that museums and schools form a common ground. As a consequence, the majority of the Czech population does not have much direct experience with these methods from their own school attendance.

The transfer of the constructivist approach from the school to the Czech museum environment brings many changes for various reasons. For example, the position of museum educator was officially established in the job catalogue in 2017. So, before that museum educators had to work in other positions and museum education was just a part of their job description [9]. While the profession is quite new in the Czech Republic, it is significantly developed and supported by the community-driven exchange of ideas and approaches (ibid.). For Czech teachers, the main dimension of constructivism is critical thinking, which consists of various thinking processes such as analysis, synthesis and evaluation [7]. This corresponds to the need to promote interpretation and dialogue among visitors [10]. In the specific case of family visits, it means that adults play a crucial role in facilitating exploration by observing their children’s interests and providing support and guidance when needed. Effective explanation by parents is important for children’s exploratory behaviour [11]. The constructivist museum is understood as a place where situations are created in which the family is given the opportunity to (re)construct knowledge. Through dialogue, collaboration and the provision of first-hand experiences, the family has the opportunity to create, modify and improve their existing knowledge. Positive parent-child interactions are essential for a meaningful museum experience, while child-led exploration and activity are also very important [12]. This approach encourages curiosity, autonomy and deeper engagement with the exhibits.

However, constructivist didactics in museums have many different aspects and, in this context, the research focuses on exploring the level of children’s activity as part of a student-centred approach. This corresponds with the aim of discovering the extent to which children are able to participate in decision-making [13,14]. Dialogue and collaboration alongside play and exploration as key aspects are examined according to the specific design of the exhibition area.

## Methods

### Study setting

This paper presents results of an observational study carried out in the Children’s Museum of the National Museum in Prague. The National Museum (NM) is the largest museum in the Czech Republic, with several partly-independent branches in Prague and other places outside the capital. Founded in 1818, it has been exhibiting Czech cultural heritage for over 200 years. The NM’s historical building in Prague recently underwent a major renovation, which concluded with a partial reopening in October 2018. The renovation provided a unique opportunity for the museum to reflect on its historical, contemporary and future role in the public sphere. Plus, the museum management had the opportunity to create new exhibitions that reflect current trends in the presentation of cultural heritage. The NM is an important destination for foreign tourists, so the permanent exhibitions have been designed around broadly understandable themes such as nature, evolution or animals, rather than specific historical themes that are more easily understood by local people. As a result of the renovation, a new “Museum complex” was created by connecting the Historical and the adjacent New Building of NM using an underground passage with a multimedia exhibition. The New Building, formerly the seat of the Czechoslovak parliament, was acquired by NM in 2009. It was built in 1974 in the brutalist style (with strong influences from the so-called Brussels style). This complex offers visitors a wide range of permanent and temporary exhibitions, rest areas, museum shops and other services such as cafes.

To address a relative lack of consideration for children and families in the NM exhibitions, the museum management decided (the initiative came from the NM’s then deputy director, Michal Stehlík, who supervised the ChM project) to create a dedicated Children’s Museum (ChM). The ChM is located in the New Building with spaces that differ significantly from the exhibition areas in the Historical Building. These conditions (such as large, mirrored windows along the whole length and height of one side of the exhibition area; high ceilings; and the fact that the whole building, which was not originally designed to host exhibitions, as well as its interior decor are listed as protected monuments) have influenced exhibition design and opportunities for visitor interaction. ChM was created over several years, including during the COVID-19 pandemic period, with the core of the team coming from the NM’s educational department. This is rather rare in Czech museum practice, as the main authors of exhibitions are typically curators: persons who are content specialists rather than educators who are more visitor- and experience-oriented. The ChM team changed over the years and cooperation with artists, graphic designers and several contractors also shaped the creative process.

The exhibition concept emerged from modern pedagogical approaches, which will be discussed later, and the team took into account several similar children’s museums in nearby cities (Futurium in Berlin, Kindermuseum in Vienna, ANOHA in Berlin and Czech science centre franchises such as IQLandia). The ChM officially opened on 30 June 2023.

Within the organisational structure of the NM, the ChM ranks among the permanent exhibitions in the museum complex. However, from the outside, the ChM stands out from other permanent exhibitions: not only because of its name. Unlike the permanent exhibitions, the ChM is not accessible with the general museum complex admission and requires a separate ticket. Tickets to the ChM are only valid for 90-minute slots. Four slots are available daily at 10:15, 12:15, 14:15, and 16:15, with a maximum capacity of 120 visitors per slot.

In addition to its own admission system, the ChM differs from other exhibitions by the presence of facilitators. The facilitators’ key task is to assist visitors with orientation in the exhibition: both in terms of navigation and conceptually. They are supposed to provide hints (to both children and adults) about the exhibition’s central idea (the thematic division) and direct visitors’ attention to visual clues in the exhibition so those persons can ideally discover the idea themselves. Facilitators are instructed not to intervene when visitors interact with the exhibition without apparent problems. Besides that, facilitators also have a traditional custodian role, taking care of visitor safety as well as the exhibits. During each slot, four facilitators are present in the ChM, covering most, but not all, of the exhibition space.

The central concept of the exhibition is “learning about the world from a different perspective than provided by the school curriculum”. The central exhibition hall is designed to have an overall museum-like feel to visitors (i.e., the space is filled with showcases and free-standing exhibits as in a “normal” museum, but the contents of the showcases and the functionality of the exhibits are non-trivial – they represent the exhibition’s 10 themes. Plus, the division of the themes is not immediately obvious.

The exhibition contains both display cases with exhibits and free-standing exhibits and interactive elements – both mechanical and electronic.

The ChM presents ten themes: each designed to offer a unique way to explore the complexity of the world around us. These themes deliberately transcend traditional disciplinary boundaries, merging natural and physical sciences with historical and cultural perspectives. Rather than adhering to conventional categorisations like “natural history,” “geology,” or “art history,” the selected themes form a mosaic of interdisciplinary inquiry. Each theme represents a distinct area of knowledge, yet they collectively provide a holistic framework that integrates both STEM and humanistic perspectives. This approach not only enables a more comprehensive exploration of the subjects, but also encourages critical thinking and curiosity as visitors are invited to draw connections across traditionally separate fields of study.

Themes are not explicitly identified for the visitors. The only identification of the thematic units within the exhibition is via symbols, colour themes and evocative questions used in the exhibition graphics. Table 1 below provides an overview of the themes.

**Table 1:** Topics and evocative questions.

Theme	Symbol	Evocative question
*Shine*	star	What do magpies and humans find attractive?
*Play*	bricks	What do we enjoy at any age?
*Error*	?!	What do we learn from?
*Fragility*	shards	Butterfly wings, dry leaves and peace – what do they have in common?
*Protection*	×	What does a ram have horns for and a gazelle legs?
*Shelter*	wigwam	Where do you lay your head?
*Motion*	arrow	Without what does a shark drown and a person grow lazy?
*Senses*	eye	So many gardens, so many fences, so many heads, so many…?
*Evolution*	crystals	What never stops?
*Record*	gramophone record	What do a trilobite and a diary have in common?

Each theme has a dedicated display case with exhibits arranged in a way reflecting the blurry boundaries between the theme’s natural/physical and historic/cultural aspects. For every theme, there is also at least one interactive exhibit and typically one non-interactive free-standing exhibit.

The exhibition contains a relatively small number of textual clues. The thematic units in the exhibition are not explicitly presented: interpretive initiative is left to visitors, who are invited to find their own way through the museum. It is assumed that the main exhibition hall’s layout and aesthetic impression will naturally evoke in visitors the idea of some internal structure. However, it will not be obvious at first glance what exactly this consists of. The ChM’s on-site staff (facilitators, see below) encourage the use of collaborative activities and help visitors to navigate through the exhibition and to convey its message. Secondly, they introduce the themes themselves in a non-intrusive, rather evocative way and help children to find the internal logic of the exhibition and to look for connections between the exhibits.

The ChM’s themes are signalled visually by symbols and colours and textually by evocative questions. These three identifiers are placed on the thematic display cases, and all free-standing exhibits and other objects are also labelled with a thematic symbol.

### Objectives

The aim of this study is to explore the alignment of the ChM’s concept and educational aims with its actual visitor experience. Specifically, we look to identify what visitor groups are attracted to at the ChM and to understand the specifics of their engagement within the exhibition. Our general research questions focused on i) the composition and other characteristics of the visiting families, ii) who is active during the visit and iii) how visitor activity varies across exhibition and iv) how the constructivist principles might affect visitor engagement.

We carried out an observational study to address these questions. Observational research on (child) visitor engagement and interaction in museums has increasingly focused on how exhibit design and social dynamics shape participation. Block et al.’s [15] study is an example of a quantitative assessment of group characteristics influencing engagement with exhibits in a science museum measured by a combination of physical, verbal and affective behaviours. Shaby et al. [16] examined science museum exhibits and identified design features that encourage sustained student involvement using Barriault’s Visitor Engagement Framework [17] for qualitative assessment of “initiation”, “transition” and “breakthrough” observable behaviours constituting visitor engagement. Mast et al. [18] investigated participation patterns in playful interactive exhibits through situated observations, proposing the Participant Journey Map as a tool for analyzing engagement over time. Lukes et al. [19] investigated visitor interaction with the help of standardized observation protocol [20] that captured measures such as group size, inferred visitor age or participant engagement and behaviour (e.g., enjoyment, boredom or communication).

Our study aims to capture a broad spectrum of interaction behaviours across multiple selected points within the exhibition. To achieve this, we adopted a standardized, protocol-based observation approach. In addition to these structured observations, we recorded extensive qualitative notes. In this paper, we focus primarily on reporting the quantitative findings derived from the protocol-based observations.

The basic unit of observation is described here as “family” – not necessarily a group of relatives, rather any group of children and accompanying adults that enter the ChM together. School tours and other larger organised groups of visitors were not the focus of this study.

In order to capture the diversity of the content and types of visitor experience in the ChM, five observation units were selected across the exhibition (Table 2). The choice of the units reflected the exhibition’s spatial organisation and its thematic structure as well as the different interaction principles expected to come to the fore based on the exhibition design.

**Table 2:** Observation units.

Observation unit	Description	Theme
U1: Wall	The interactive wall is the first feature of the ChM that visitors encounter upon entry. This interactive installation covers the entire wall to the right of the entrance. It is based on video mapping combined with LIDAR technology [21,22]. On the wall, a series of 10 mini-games is projected. They are controlled by throwing Styrofoam balls. Each game is dedicated to one of the exhibition’s themes and allows for engagement by multiple players who collaborate to finish the game before time runs out; e.g., the theme *fragility* is represented by a game in which players put together pieces of broken ceramics. The unit area offers seating covered with evocative questions.	All 10 themes (see Table 1).
	U2: Knight		The “Knight” is a freestanding sculpture representing a life-sized figure of an imaginative knight. It is entirely composed of animal shells, carapaces and armours as well as “protective” parts of plants such as thorns, spikes and nut shells. For instance, the swordfish’s bill represents the knight’s sword, and the blowfish’s skeleton symbolises the knight’s head. The composition resembles Giuseppe Arcimboldo’s paintings. The sculpture is placed in a glass case and can be viewed from all sides. Each side of the display case features a transparent outline graphic identifying the major segments of the knight’s armour. The object is connected with an adjacent showcase with items related to the topic as well as evocative questions and labels.	*protection*
U3: Theatre		The theatre is divided into two tables. The first table serves as a mirror stand where all the wooden masks are placed. Each wooden mask represents a different emotion, and visitors can choose from a variety of faces or from the descriptions written on the handle. The other table is the actual theatre set, where visitors can let their imaginations run wild and play as they please. Underneath the stage, there are drawers full of puppets and scenery. The accompanying showcase with historic toys helps boost visitors’ engagement.	*play*	
	U4: City	The display’s full name is “City through the ages”. It is located upstairs in a separate room and is an open space playground with the main aim of building your own city based on the visitor’s personal choice. The room consists of carpets with city designs, bean bags and shelves with building blocks with different designs based on a particular architectural period. Visitors can find out more from the board on the wall where all the architectural types are grouped according to era and colour.		*evolution*
U5: Hive		Before they leave the exhibition, visitors may stop at the last hall: the “Hive”. Equipped with hexagonal tables and chairs and hexagonal built-in closets, this hall’s overall style evokes a beehive. This is also supported by the design of the original hexagon-shaped ceiling. The Hive is primarily designed for creative and group activities. In the built-in closets, visitors can find thematic books, games and worksheets as well as tools for creative activities. One worksheet is available for each of the exhibition’s 10 themes – e.g., for the theme *protection*, there is a worksheet on making animal armour from walnut shells.	All 10 themes (see Table 1)	

### Observation procedure

The observation process started on 16 March and ended on 30 May 2024. The observations were carried out by 19 observers – mostly students of a Museum Studies master’s programme who were trained in systematic observation techniques during a workshop led by an expert ethnologist. The workshop introduced principles of ethnographic observation grounded in interpretive ethnography, emphasizing attention to interactional dynamics and meaning-making *in situ*. Observers were assigned to a particular observation unit.

For the standardized part of the observation, an observation protocol was developed following structured ethnographic guidelines [23]. It contained scalar items dedicated to visitor interaction and engagement (addressed below) as well as a number of other measures. These included the circumstances of the visit (day of the week, time slot, perceived crowdedness, and noise level measured with a smartphone app), the composition of the visitor group (age cohort and gender), the degree of engagement with labels, symbols, and evocative questions, and the dwell time. The protocols, tailored to specific units, also included a schematic plan of the observation area, allowing observers to sketch a simple diagram of the group’s movement.

In addition to the protocols, observers were instructed to take open-ended field notes to capture contextual details and interpretive insights beyond the standardized items [24] and to keep a field diary for overall commentary and to summarize the observed patterns. The extensive field notes, complemented by area diagrams, will be analyzed separately in a subsequent qualitative study. Below, only a few preliminary observations from the field notes are provided to support the quantitative findings. After the first week of observations, the protocol was revisited to better reflect actual visitor behaviour. To ensure the consistency of the observations across units and individual observers, periodical feedback sessions with the entire team were held. A senior member of the team (one of the authors) contributed to the data collection at each unit.

Typically only one observer was present in the ChM during a visitor slot. They were stationed at a single observation unit. Observers did not interact with the visitors or the ChM facilitators in any way. Although visitors were not explicitly informed about the observation process as no personal or sensitive information was collected, the act of observation was not disguised. Upon inquiry (which was seldom), observers did reveal the purpose of their presence. Otherwise they were likely perceived as museum employees (as evidenced by some visitors confusing observers with facilitators). The procedure can thus be described as non-participant and implicitly overt (or semi-covert [25]).

### Observed phenomena

In the current study, we focus on measuring visitors’ interaction with the exhibition and their overall engagement as well as measuring visitors’ reactions to textual and image information in the exhibition.

#### Interaction.

According to the designers of the ChM, the exhibition’s concept reflects three general principles of interaction between the visitor and the exhibition: learning, playing and creating (https://www.nm.cz/en/program/families-and-children/childrens-museum). We took these three principles as a basis for our observation of visitor behaviour. We understand the playing interactions to be more physical; with visitors adhering to given rules, or creating their own negotiating roles in the game, or competing [26]. It is important to note that not all play is always obvious or visible. People may engage in solitary play [27], such as daydreaming, which may not be immediately apparent to an observer. Learning, on the other hand, involves analytical processes: such as asking and answering questions [28], meaning-making [29], decoding purposes and creating relationships between displayed objects [30]. Finally, creating centres on producing something new (writing, drawing, building, etc.), inspired by the museum’s content and developing it further.

The three principles of interaction envisaged by the authors of the ChM were operationalised here as aggregates of specific behaviours (interaction dimensions) that can be assessed during observation by using a simple scale. Rather than assuming the three principles to be discrete categories, we adopted a multidimensional perspective with overlapping scales. This is because some observable behaviours may be characteristic for multiple types of interaction at once. Table 3 provides an overview of the measured dimensions of learning, creating and playing together with the simplified operationalization.

**Table 3:** Dimensions of learning, creating and playing.

Dimension	Learning	Creating	Playing	Manifested behaviour
*Focus*	✓	✓		Silence, being absorbed by the activity, calm and concentrated engagement
*Confusion*	✓			Furrowed brow, puzzled expression, hesitation in speech, searching for words, fidgeting etc.
*Frustration*	✓	✓	✓	Discontented expression, raised voice, swearing, angry gesticulation etc.
*Communication*	✓	✓	✓	Asking and answering, reassurance, nodding, repeating, pointing etc.
*Explaining*	✓			Parents explaining to children, children explaining to each other etc.
*Creating*		✓		Drawing, writing, building, explaining, cutting, gluing, using tools and materials etc.
*Problem-solving*		✓	✓	Naming the problem and finding a solution, asking for help (adult, facilitator, child, stranger) etc.
*Cooperation*		✓	✓	Doing things together, sharing common ground, joint attention, negotiating rules etc.
*Imagination*		✓		Reworking stories, giving new names, inventing situations, daydreaming etc.
*Motion*			✓	Jumping, running, pacing, standing up, lying down etc.

The procedure leading to the selection of interaction dimensions involved:

(i) identifying of concepts related to learning, creating and playing in the literature;
(ii) pre-selecting concepts based on the exhibition’s affordances and operationalizing them for observation;
(iii) testing the concepts through pilot observations and finalizing the set of measured items;
(iv) conducting *post-hoc* analysis to assess how the data fit the proposed variable structure.

Each item was measured on a 4-point Likert scale (0–3). *Post-hoc* analysis revealed that the interaction dimensions were not substantially intercorrelated, which supports the decision to analyze them individually rather than as indicators of latent constructs.

Our approach diverges from previous research by focusing on the visitor group as a whole rather than individual participants. Using a complex observation protocol, we combine qualitative data (with field notes and simple maps similar to [18] and quantitative data – expanding the range of observable phenomena captured compared to previous studies [15,16,19].

#### Engagement.

Observers assessed the visitors’ overall engagement (children and adults separately) and the work of museum facilitators. Again, a 4-point Likert scale was used (with 0 = no engagement). For all groups, engagement was defined as “the degree to which [observed subjects] are engaged in the observed interaction”.

Whereas engagement of the children as well as their adult company was expected, facilitators did not necessarily have to enter the interaction.

#### Visual and textual cues.

Observation involved also the measure of visitors’ reactions to visual information present in the ChM: evocative questions, symbols and colours that encode the exhibition’s themes and labels on the exhibits and other texts. The scale was the same as for interaction and engagement (0–3). Observed visitor behaviours of course reflect only partially these persons’ awareness of visual information – the measures reported here capture visitors’ visible reactions related to noticing information: pointing, metacommunication or other manifestations of joint attention directed towards it.

## Results

### Capacity

The 2.5 month observation period yielded 720 observations (the data and R scripts can be found at: https://osf.io/vm9ra). The observers’ fieldwork in the ChM was scheduled in order to cover the changing nature of visits over the week and during each day’s four visitor slots. Observations were distributed evenly across U1–U5 (see Table 4).

**Table 4:** Number of observations across units.

Unit	Observations
*U1*	158
*U2*	144
*U3*	156
*U4*	125
*U5*	137

As the number of visitors fluctuates during the week, peaking on weekends, it was important to ensure that the observations were not skewed towards certain parts of the week and that they captured the diversity of visitor experiences related to perceived crowdedness. Table 5 shows the number of observations on each day of the week as well as the relative proportion of visitors captured by the observers.

**Table 5:** Overview of the observations.

Day	Observations	Avg. capacity	Visitors observed	% of all visitors
*Mo*	59	0.34	194	0.23
*Tu*	93	0.39	267	0.24
*We*	92	0.50	265	0.18
*Th*	49	0.33	157	0.22
*Fr*	41	0.51	125	0.14
*Sa*	224	0.76	691	0.28
*Su*	162	0.75	474	0.20

During the observation period, weekends were the busiest days at the ChM (average capacity 0.76). On workdays, Mondays, Tuesdays and Thursdays were the least busy (average capacity 0.35), with a mid-week peak on Wednesdays (0.50) and a “pre-weekend” effect on Fridays (0.51). Observers managed to capture a stable proportion of visitors on each weekday (average 0.23); except for Friday which had a relatively lower proportion (0.14).

The first visitor slot is on average the busiest, while the last late afternoon slot has the lowest average number of tickets sold. The distribution of observations across slots reflects this tendency. Table 6 shows that the relative frequency of visitors in the particular slots matches the relative frequency of observations.

**Table 6:** Frequency of observations and capacity across slots.

Slot	Avg. capacity	Proportion of visitors	Proportion of observations
*1*	0.63	0.33	0.30
*2*	0.50	0.25	0.27
*3*	0.51	0.25	0.27
*4*	0.34	0.17	0.15

### Visitors in the ChM

In total, 2173 visitors were subjected to observation. Typically, there was single observer present in the ChM. Therefore, the vast majority of the individual visitors were not observed repeatedly. However, there were cases when multiple observers were collecting data at different units during the same slot. These observations involved 414 (19% of the total) subjects who could have potentially been observed more than once during their visit. In the analysis of the visitor group composition reported below, these observations are discarded (n_(after data reduction)_ = 1759), in order to report the number of individual visitors. For the visitor behaviour analysis, these 414 observations will not be discarded, as the basic unit of observation is a particular visitor group present at a particular observation unit – and in this regard, all observations are unique and independent.

Table 7 shows the gender and age distribution of the visitors grouped in age cohorts (based on observers’ estimations). The groups reflect the Czech educational system. Elementary education spans nine years, typically for children aged 6–15, divided into First Stage (Grades 1–5) and Second Stage (Grades 6–9). Toddlers = 0–3; preschoolers = 4–6; junior primary school pupils = 7–10 (~ First Stage); senior primary school pupils = 11–15 (~ Second Stage); teenagers, secondary school students = 15–19; young accompanying adult = 20–35; middle-aged accompanying adult = 36–59; elderly accompanying adult = 60 + .

**Table 7:** Visitors – age and gender distribution.

	Toddlers	Preschoolers	Junior primary	Senior primary	Teenager	Young adults	Middle-aged	Elderly
*F*	120	248	120	32	1	221	279	62
*M*	87	196	87	9	1	87	190	19

**Fig 1:**
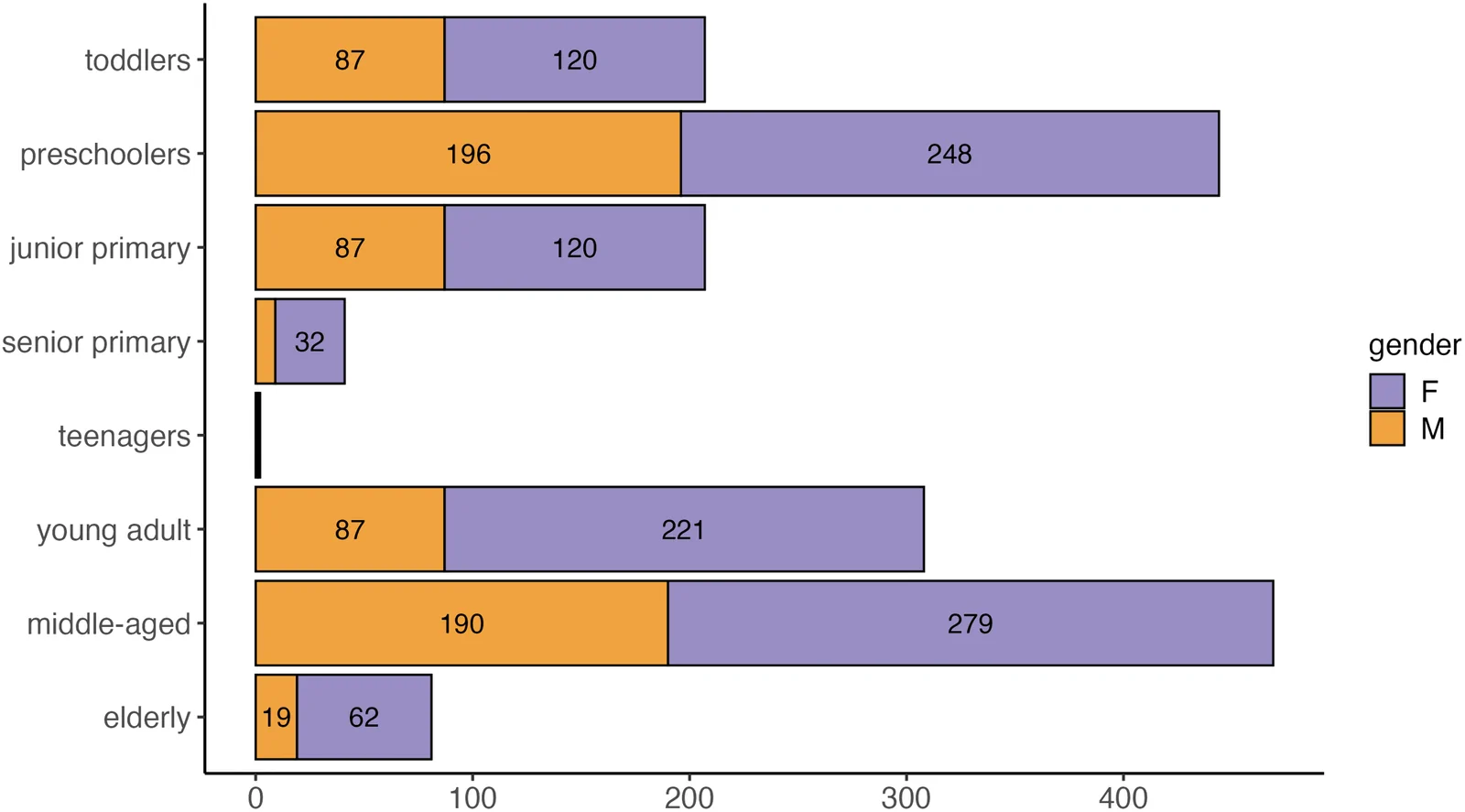
Visitors – age and gender distribution.

Across age groups, female visitors predominated (F = 61.6% vs. M = 38.4%). As Fig 1 illustrates, this gender imbalance was greater in the adult age groups (F = 65.5% vs. M = 35.5%), but among children female visitors were also in majority (F = 57.8%, M = 42.2%). As for 2023 (the most recent census data), the gender ratio within the Czech population was M:F = 0.96 [31]. The overall gender ratio in our sample is M:F = 0.62. The most striking imbalance was found in the group of elderly accompanying adults (M:F = 0.31), while the most balanced age group in terms of gender was the junior primary school cohort (M:F = 0.79).

The average number of visitors per observation (i.e., per “family”) was 3.06. More than one-third of visitors came in pairs (an adult and a child, n = 207, 35.75%). Three- (n = 191, 32.99%) and four-person groups (n = 133, 22.97%) followed with these three group sizes making up most of the visitors.

Looking at the composition of the visitor groups, the most frequent types of groups observed included a girl of preschool age accompanied by either a female middle-aged adult (n = 25, 4.4%) or a female and a male middle-aged adult (n = 25, 4.4%). The third most common family composition was again a preschool girl accompanied by a young female adult. In the 3-person groups, there was a slight majority of adults over children: 271 vs. 269. The specific gender and age composition of 2-, 3- and 4-person groups varied without a dominant pattern (see Supplement 1 at the OSF repository for more details). Overall, the number of child and adult visitors across age groups was also comparable, with 901 children and 858 adults.

In terms of languages spoken, 75.82% of the visitor groups were Czech. The rest of the observation consists of mostly unidentified languages (7.42%) and languages identified as English (4.32%) and Slovak (3.45%) (These cases also include mixed groups where one parent is Czech). Czech and non-Czech groups differed in terms of size (M_Czech_ = 2.99, M_non-Czech_ = 3.31, W = 24681.5, *p* < 0.001, *r* = −0.146), but their age and gender distribution was comparable.

To generalise, a typical visitor group to the ChM has 2–4 persons who are from the Czech Republic. Female visitors are a more likely sighting: both as concerns children and adults. The most typical age group for children is preschoolers, while adults are most likely expected to fall in the middle-aged category (both genders) or young-adult category (in the case of women). If there is a single adult companion, it is three times more likely to be female than male. Interestingly, elderly persons accompany children in the ChM rather rarely (and when they do come, they also tend predominantly to be women).

### Visitor activity and exhibition design

#### Dwell time.

Observers recorded the time spent at particular observation units (the vast majority of visitor groups were observed only at a single unit; thus, we cannot estimate the total dwell time for families in the ChM).

**Fig 2:**
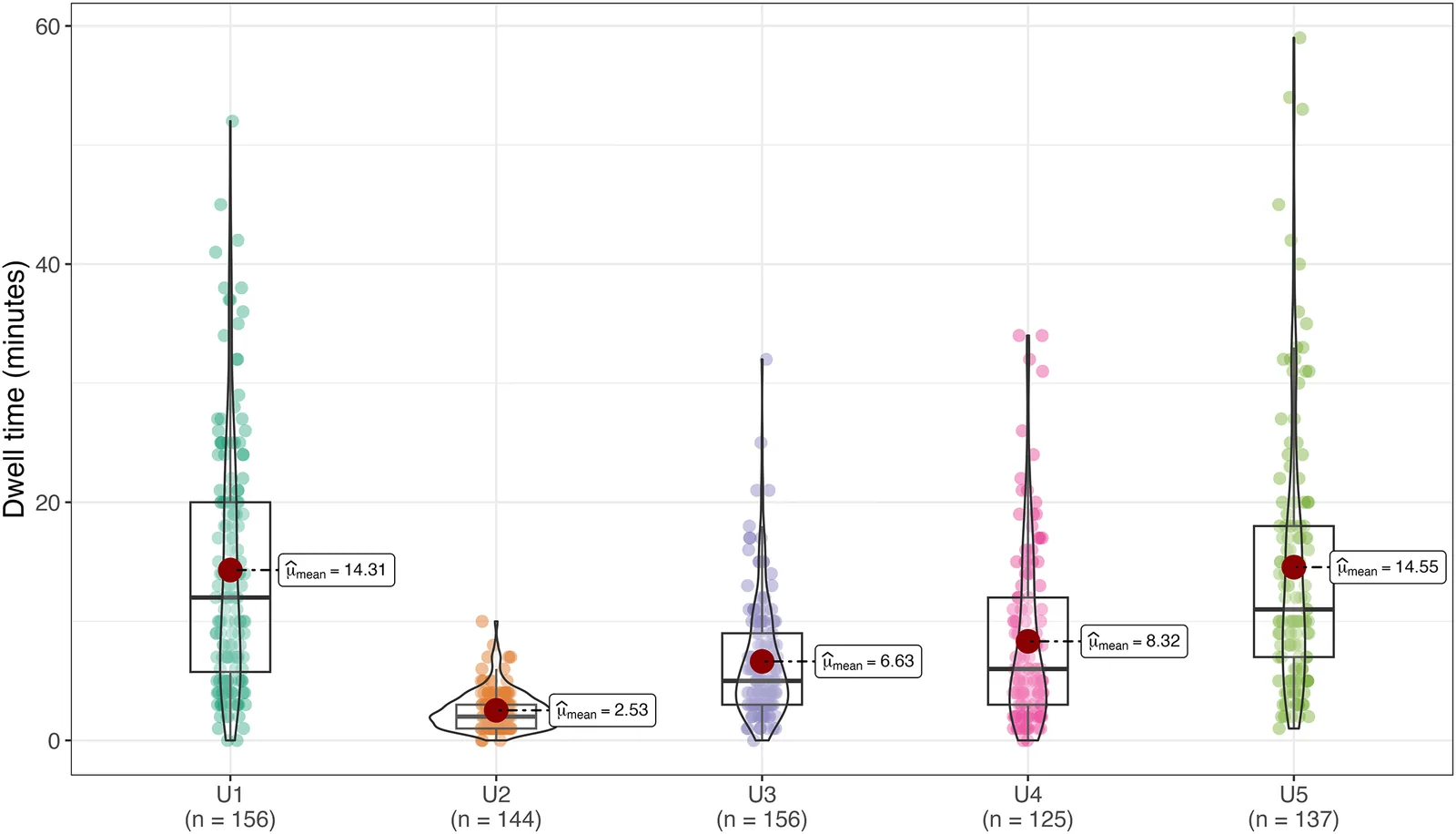
Dwell time across units, dot = mean, box = interquartile range, bar = median.

On average, visitors spent 7 minutes at a unit. However, there were considerable differences across units. The longest average dwell times were observed at U5 and U1 (14 minutes), whereas U2 had the shortest average dwell time (about 2.5 minutes). A Kruskal-Wallis test (*χ*²_(4)_ = 261.36, *p* < 0.001, *ε²* = 0.36) followed by a pairwise Wilcoxon rank-sum test revealed that U2 was the only unit that differed significantly from all other units in terms of dwell time. Fig 2 shows the mean time spent at each unit.

It is not surprising that U2 stands out in such a way. There are several reasons for this. First, unlike the other observation units, U2 does not provide any opportunity for visitors to sit down. Second, it is situated right in the middle of the main route visitors take when passing to and from the exhibition’s second floor. U1 and U5, on the other hand, represent two key “hubs” within the ChM. As a space for collaborative activities located by the exit from the exhibition, U5 is naturally where the visit concludes – with both adults and children seated at tables: often until their tickets expire. The average dwell time at U1 also includes cases where the observed group returned (total dwell time is then a sum of two visits). This was the case with half of the observations at U1. If we ignore returnees, the average dwell time at U1 drops to 11 minutes, which is still a substantial part of the running time of one series of interactive games. This means that the interactive wall succeeds in attracting visitors’ attention for a relatively long time before they proceed further into the exhibition.

Below, we proceed with the quantitative analyses of the dimensions of visitor behaviour captured by the scalar measures: types of visitor interaction with the exhibition and each other, overall levels of engagement of different types of participants, and the degree of visitors’ reactions to visual cues.

#### Interaction.

**Fig 3:**
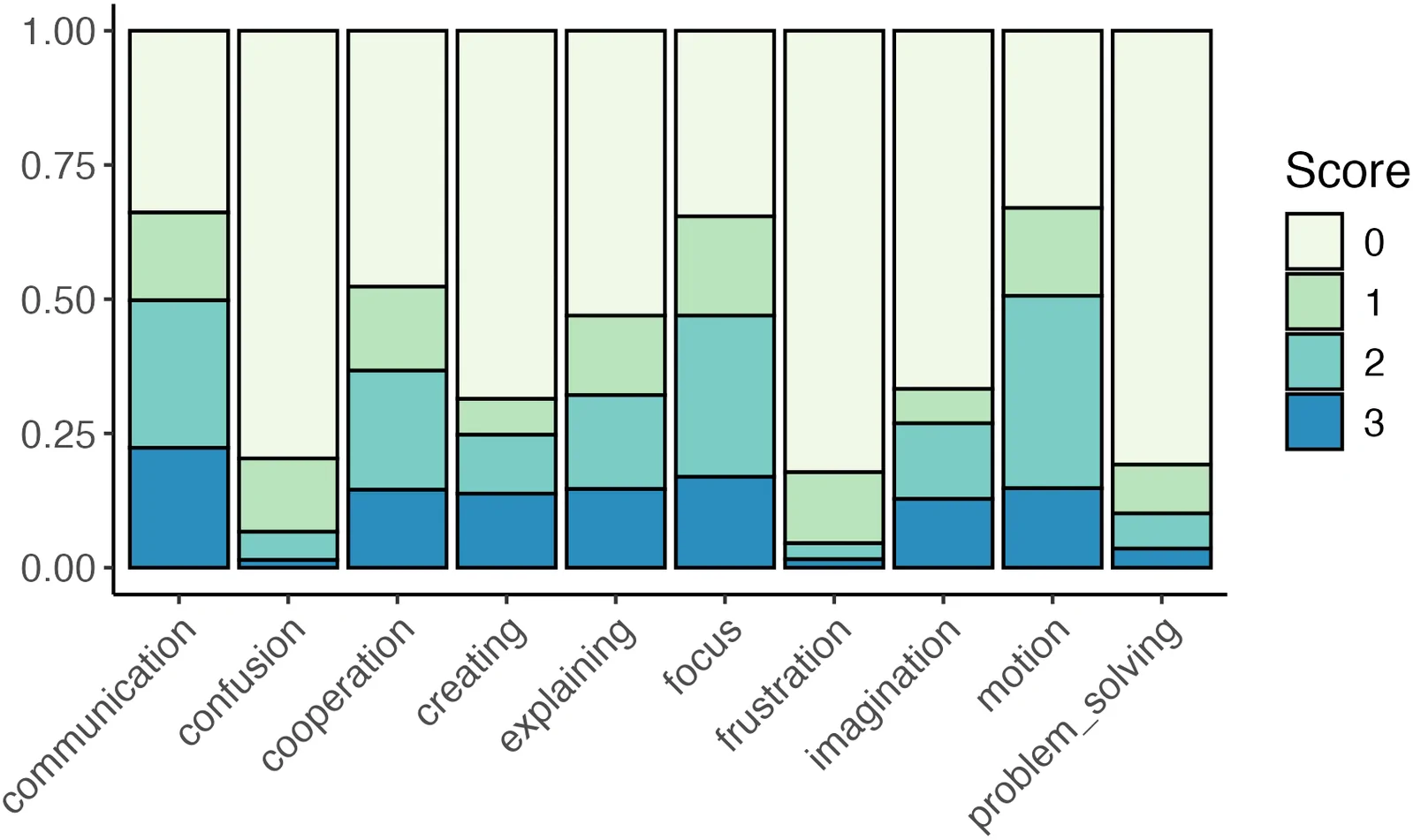
Proportions of interaction scores.

Prior to the quantitative analysis, 17 observations were excluded due to coding inconsistencies. This resulted in a final dataset of 703 observations. Table 8 summarises the mean scores for each type of interaction. Fig 3 shows the proportions of interaction scores.

**Table 8:** Mean interaction scores.

Variable	Mean
*Communication*	1.38
*Motion*	1.32
*Focus*	1.29
*Cooperation*	1.04
*Explaining*	0.94
*Imagination*	0.73
*Creating*	0.70
*Problem-solving*	0.33
*Confusion*	0.28
*Frustration*	0.24

**Fig 4:**
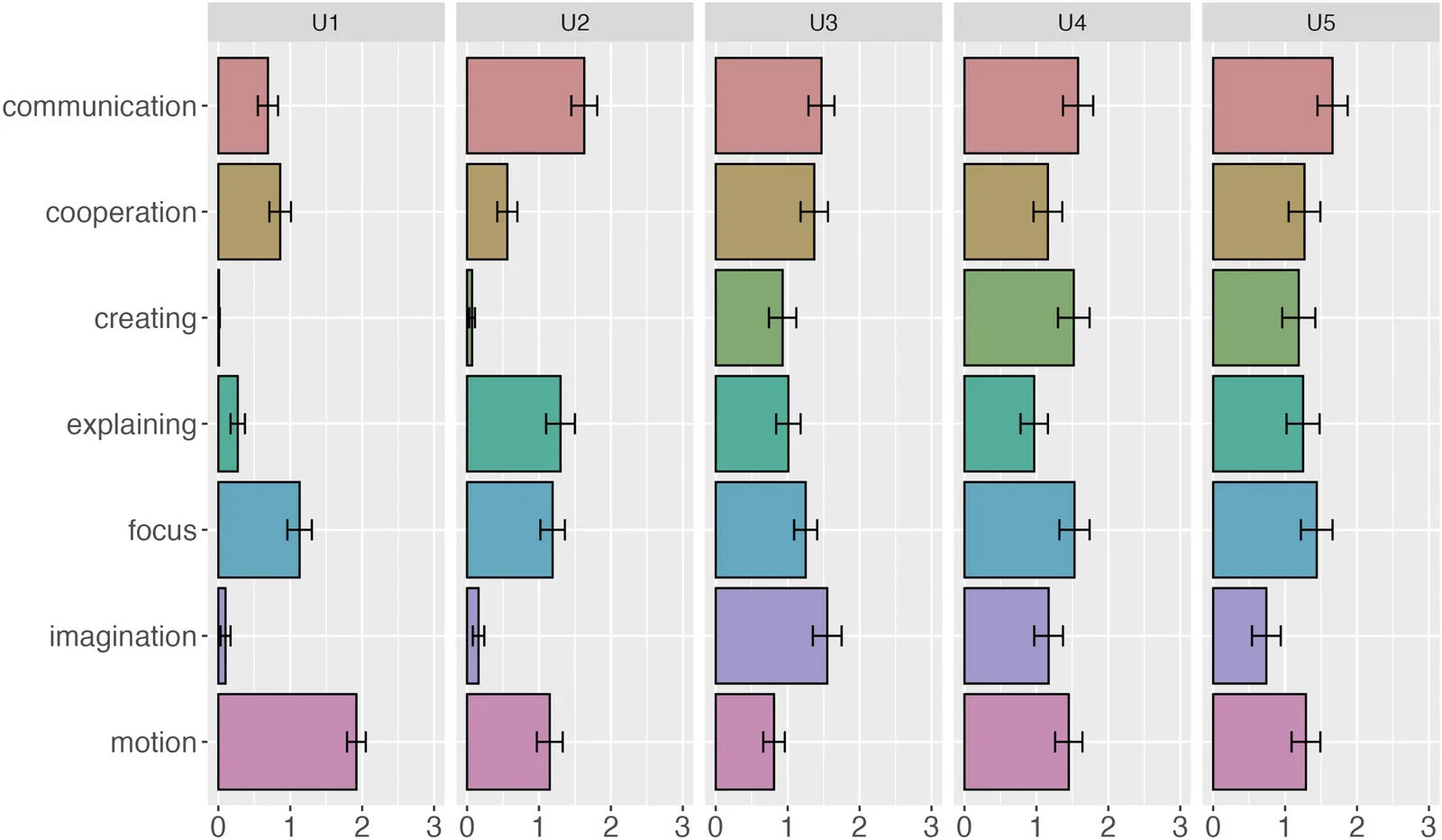
Mean interaction scores across units with 95% CIs.

Average scores for *confusion*, *frustration* and *problem-solving* did not reach 0.5 and these three interaction types were also the only ones with an average score below 1.0 in every unit. These three types explain a relatively marginal part of the overall variation within our data. Thus, we will not deal with these types in our quantitative analysis. With seven interaction types left that were not substantially intercorrelated (the highest correlation was found between communication and explaining, Pearson’s *r* = 0.58, i.e., moderate correlation), we will further treat them separately and not as part of the latent variables. Fig 4 shows the average interaction scores of the remaining interaction types across units.

We analyzed interaction scores across the seven dimensions using cumulative link mixed models for ordinal outcomes (with the help of *ordinal* package [32] in R [33]. Each interaction type was a separate response variable. Fixed effects included unit, age group, time slot, slot capacity, group size, and day of visit. Interaction terms between unit and age-group were added and a random intercept for observer was included to account for observer variance.

Observations captured interactions at the level of a visitor group (typically a family), so individual interactions could not be distinguished. We focused on the age composition of children present in each group. Ninety percent of all visitor groups fell into one of five types: groups with preschoolers only (n = 273, 39%), toddlers only (n = 112, 16%), junior-primary pupils only (n = 105, 15%), toddlers and preschoolers (n = 85, 12%), and junior- plus senior-primary pupils (n = 60, 9%). To examine the effect of age composition, we included these five types as a categorical predictor in our models. The remaining 68 groups represented less frequent age combinations and were excluded from the analysis.

**Interactions across units:** Ordinal regression models (see full results in Supplement 2 at the OSF repository) revealed substantial differences between units in the likelihood of eliciting specific interaction types. Compared to U1 (reference), U3 and U5 consistently showed the strongest positive associations with creativity-related interactions, while U2 occupied an intermediate position.

- *Focus*: U5 had the highest odds of focus-related interactions (odds ratio (OR) = 14.33, 95% confidence interval (CI) [2.98, 68.95], *p* = .001), followed by U4 (OR = 9.86, 95% CI [1.30, 75.12], *p* = .027) and U3 (OR = 6.35, 95% CI [0.95, 42.45], *p* = .057). U2 showed no significant difference (OR = 1.73, 95% CI [0.28, 10.66], *p* = .556).
- *Communication*: U3 was strongly associated with communication (OR = 9.33, 95% CI [1.88, 46.17], *p* = .006), while U2 and U5 showed moderate, non-significant increases (OR ≈ 3.8–3.9, CIs overlapping 1).
- *Creating*: Creativity-related interactions were dramatically more likely in U3 (OR = 178.86, 95% CI [10.96, 2917.59], *p* < .001), U4 (OR = 594.36, 95% CI [34.31, 10296.70], *p* < .001), and U5 (OR = 401.23, 95% CI [25.62, 6284.75], *p* < .001), compared to U1.
- *Explaining*: No unit showed a significant effect, with U5 exhibiting the highest odds (OR = 4.39, 95% CI [0.81, 23.90], *p* = .087).
- *Imagination*: U3 and U4 exhibited very strong associations (OR = 177, 95% CI [19.56, 1601.42], p < .001; OR = 57.26, 95% CI [6.11, 536.54], *p* < .001), with U5 also significant (OR = 13.57, 95% CI [2.90, 63.48], *p* = .001).
- *Cooperation*: No significant differences emerged across units (ORs close to 1, wide CIs).
- *Motion*: U2, U3, and U5 were associated with substantially lower odds of motion-related interactions compared to U1 (ORs = 0.11–0.04, 95% CIs well below 1, *p* ≤ .01).

**Interactions and age groups:** Except for *creation*, which has overall lower odds with the toddler-preschooler groups (OR = 0.4, CI [0.17–0.94], *p* = 0.036), the age group modifies the effect of interaction dimensions on *focus*, *explaining*, and *communication* only in certain units.

- Pairwise contrasts indicate that, for *focus*, age profiles generally do not differ significantly within units, except in U5 where groups with junior-primary cohorts have significantly higher predicted *focus* scores than toddlers (estimate ≈ 2.00, *z* = 2.9, 1 *p* ≈ 0.04).
- For *explaining*, the differences pertain to U4 and U5. In U4, junior-primary groups have significantly lower predicted probabilities compared to both preschooler (−2.29, *z* = −3.15, *p* = 0.014) and toddler-preschooler groups (−2.66, z = 0.820, *p* = 0.010). In U5, toddler-preschooler groups have significantly higher *explaining* scores compared to toddler-only groups (2.86, *z* = 3.36, *p* = 0.007).
- For *communication*, the effect of age groups was detected at U3, where junior-primary groups have greater predicted probability than toddlers (2.63, *z* = 2.961, *p* = 0.026) and U4, where junior- & senior-primary combined groups have greater predicted probability than toddler groups (3.44, *z* = 3.178, *p* = 0.013).

**Other effects:** While there was no effect of perceived crowdedness (capacity) on interaction scores, an effect of slot was detected. Compared to the first slot (reference level), the estimates for *focus*, *creating*, *imagination* and *cooperation* were significantly lower in one or several of the later slots. *Cooperation* scores were also significantly predicted by group size (OR = 1.24, CI [1.02–1.51], *p* = 0.033) and the effect of the day of the week (in comparison to reference level = Monday) was generally not significant except for the lower odds for *cooperation* on Wednesdays (OR = 0.33, CI [0.13–0.87], *p* = 0.025).

**Observer effects:** Most (14 out of 19) observers were assigned to a single unit. Thus, random intercepts for observers were partly confounded with unit variation. To assess observer bias, we compared intraclass correlation coefficient values (ICCs) from models with and without fixed effect of unit. ICC represents the proportion of variance attributable to observer differences. When unit was included, ICCs ranged from 0.18 to 0.50, indicating moderate clustering by observer. Removing unit increased ICCs substantially for some dimensions (e.g., *cooperation* from 0.24 to 0.66; *imagination* from 0.50 to 0.69), suggesting that much of the apparent observer effect was actually due to unit-specific factors. In contrast, ICC changes were minimal for *focus*, *creating*, and *motion*, implying that observer-related variability in these dimensions is more genuine and less confounded by unit.

#### Engagement.

**Fig 5:**
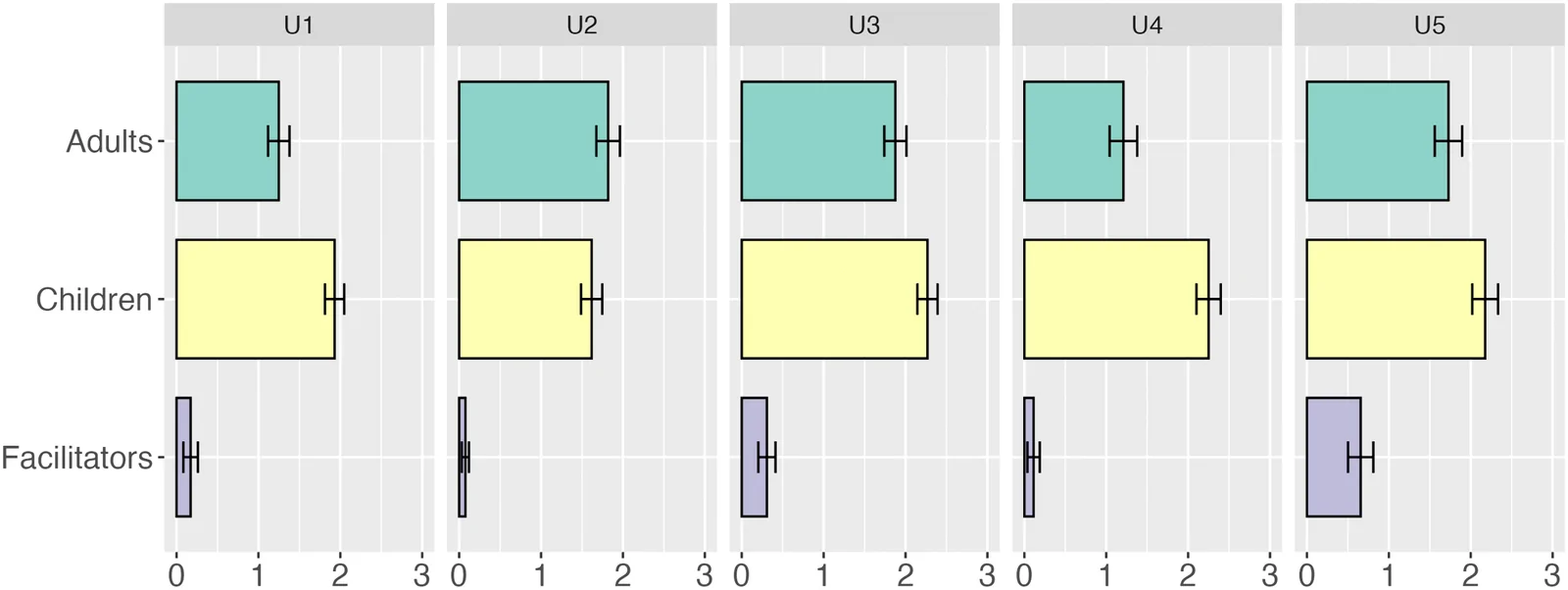
Mean engagement ratings across units.

As expected, the highest overall engagement was observed in children (2.06, SD = 0.83), with adults scoring on average lower (1.60, SD = 0.93). Facilitators’ engagement was very low (0.27, SD = 0.63). The average engagement scores across units are shown in Table 9 and Fig 5 below.

**Table 9:** Mean engagement ratings across units.

Unit	Children	Adults	Facilitators
*U1*	1.93	1.25	0.17
*U2*	1.64	1.85	0.08
*U3*	2.27	1.88	0.35
*U4*	2.27	1.22	0.11
*U5*	2.23	1.79	0.67

Separate ordinal regression models were fitted for children’s, adults’, and facilitators’ engagement scores, with the same sets of predictors as in the case of interaction dimensions. Full results of the models can be found in the Supplement 2.

**Children:**

- Engagement was significantly lower in unit U2 (OR = 0.19, CI [0.04–1.00], *p* = 0.049
- Age group interacted with unit, with higher engagement for older children compared to toddlers in some units:
  - U3: junior primary vs. toddlers (predicted probability estimate = 2.63, *z* = 0.890, *p* = 0.026)
  - U4: junior–senior primary vs. toddlers (estimate = 3.44, *z* = 1.08, *p* = 0.013)
- Adult activity was a strong positive predictor (OR = 18.09, 95% CI [10.40–31.48], *p* < 0.001)
- Larger group size was associated with higher engagement (OR = 1.30, 95% CI [1.05–1.60], *p* = 0.015).

**Adults:**

- Engagement was significantly lower in unit U4 (OR = 0.18, CI [0.04–0.74], *p* = 0.017)
- Age group interacted with unit, with higher engagement for toddler-preschooler groups in U4:
  - junior primary vs. toddler–preschooler (estimate = –2.70, *z* = −3.69, *p* = 0.002)
  - preschooler vs. toddler–preschooler (estimate = –1.79, *z* = −2.80, *p* = 0.041)
- Engagement was also lower in slot 3 (OR = 0.57, 95% CI [0.37–0.90], *p* = 0.015).

**Facilitators:**

- Engagement was markedly higher in unit U5 (OR = 15.04, CI [2.89–78.33], *p* = 0.001)
- Slot and day of the week had strong effects: engagement tends to be lowest in slot 4 (OR = 0.14, CI [0.05–0.43], *p* = 0.001) and during weekends, particularly Saturdays (OR = 0.02, CI [0.00–0.06], *p* < 0.001). Mid-week sessions on Wednesdays also showed reduced engagement (OR = 0.18, CI [0.04–0.89], *p* = 0.035).

**Observer effect:** Observer variance is highest for the facilators’ engagement, where ICC remains high and stable after adjusting for unit variation (around 0.70). For the childrens’ and adults’ engagement scores, much of the apparent observer effect is actually unit-driven.

#### Evocative questions, symbols and labels.

**Fig 6:**
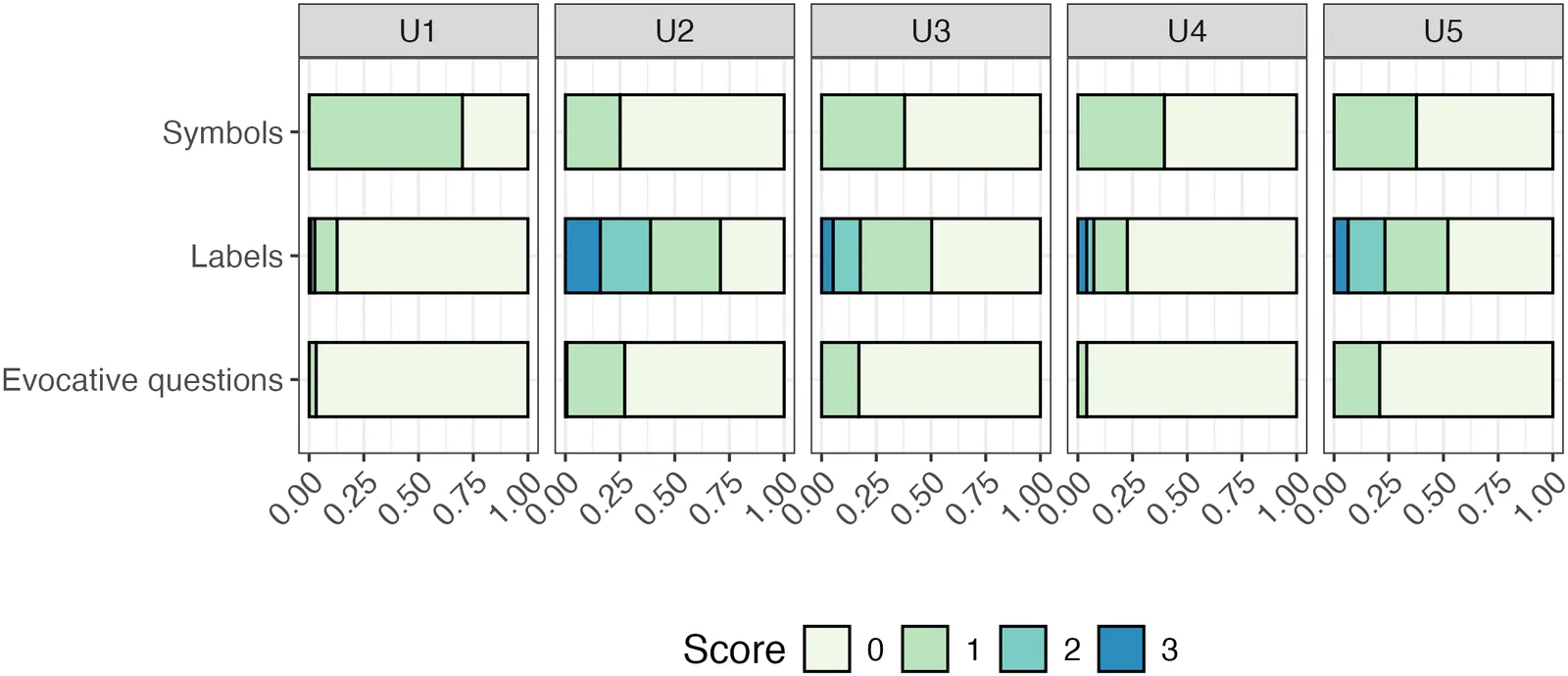
Proportion of reaction scores across units.

Observers rarely noted visitors’ reactions to either evocative questions (mean score = 0.15) symbols (0.43) or labels (0.66). Fig 6 shows the proportions of information-related scores across units.

The extent to which visitors responded to visual information varied significantly across the different units (as indicated by the Kruskal-Wallis rank sum test followed by post-hoc pairwise comparisons using the Wilcoxon rank sum test with continuity correction.

- For symbols (*χ*²_(4)_ = 69.675, *η*² = 0.094, *p* < 0.001), U1 elicited significantly more reactions compared to all other units.
- For labels (*χ*²_(4)_ = 141.18, *η*² = 0.197, *p* < 0.001), U2 was characterised by more reactions than the other units, whereas U1 and U4 were associated with significantly less reactions compared to the rest.
- For the evocative questions (*χ*²_(4)_ = 50.78, *η*² = 0.067, *p* < 0.001), U1 had significantly lower reaction scores compared to all other units.

## Discussion

The analysis revealed a predominantly female audience, with preschool-aged girls and their (also predominantly female) accompanying adults forming the largest visitor groups. This gender imbalance probably results from the fact that in the Czech Republic parental leave is longer (3 years on average) than in other OECD countries and mothers usually stay at home with their children [34]. This imbalance is also described in other studies and it is contextualised with single parenting [35] or with communication practices in specific contexts where children are more engaged in explaining things while communicating with their mothers [11].

One of the major findings is that the age distribution of child visitors is skewed towards preschoolers. This is not the primary age group for which the ChM was intended. Exhibition design is based on a constructivist approach, which relies on the ability of parents to facilitate the construction of knowledge through dialogue with their children and the use of pre-conceptions and the incorporation of new knowledge into previous knowledge. The presence of the facilitators is unique to the ChM, while in other NM exhibitions there are personnel who just provide information and are not supposed to support interaction. The research challenged the high demands placed on visitors as concerns their active attitude and it indicated that preschool children engaged less effectively with the evocative questions distributed throughout the exhibition. This is a key observation that suggests a mismatch between the exhibition’s design and affordances and the actual composition of visitor groups. The question is whether adults are able to facilitate the process of meaning making, although they themselves probably do not have much experience with this kind of constructivist museum environment. This approach to interaction is not prevalent in Czech museums nor in Czech schools. It will be worthwhile to focus on this particular aspect in further research.

Our analysis revealed significant differences between the selected observation units in terms of interaction dimensions. In general, the U3–U5 cluster stand together with a relatively higher score across interaction types. U1 has lower creativity- and communication-related interaction types than the rest, except for U2 which appears to be a kind of an “intermediate” unit. It clusters together with U3–U5 in creativity-related interactions on the one hand and with U1 in communication-related interactions on the other.

Importantly, the units differed significantly in the interaction scores across age groups. The older cohorts exhibited significantly higher creativity and communication (in U3 where interactive theatre activities encouraged complex social interactions and imaginative play and in U4) and higher focus (in U5). In contrast, the younger groups relied more on adult guidance, as suggested by higher explaining scores in U4 and U5. The older children’s behaviour appeared to be more independent and creative, whereas the younger children often relied on adult support to navigate the exhibits (as indicated in the field notes).

The analysis of interaction types revealed that communication- and attention-related behaviours were the most prevalent kinds. Problem-solving, which was not revealed as an important behaviour, is well described in constructivism and plays an important role in learning [36]. Interactions related to problem-solving are particularly associated with STEM fields or science learning [37]. ChM, however, unlike more STEM-oriented institutions like science centres, has a broader focus and thus problem-solving interaction might not come to the fore.

Some displays in the ChM, however, could be more appropriate for investigating problem-solving tasks. Moreover, expected problem-solving interactions might have been substituted by explaining and other communication-related behaviours due to the children’s young age. However, the notable differences in creativity and communication scores – particularly for U1 compared to U3, U4 and U5 – highlight the need for targeted adjustments in specific areas to enhance visitor experiences.

The model predicts higher interaction scores in the first morning slot. This pattern likely reflects age-related variations in attentional capacity across the day [38] and the cumulative effects of museum fatigue as visits progress [39]. The average size of visitor groups remains relatively stable across slots (slot 1 = 2.85, slot 2 = 3.00, slot 3 = 3.13, slot 4 = 3.18), suggesting that differences in interaction patterns are unlikely to be driven by school group size, even though early morning slots typically coincide with school visits at NM.

Children’s relatively low engagement in U2 is likely due to the fact that the main exhibit is not interactive (note that U2 also had the shortest dwell time). In U3, the higher engagement scores for older children suggest that the interactive theatre is better suited for the older cohorts, who often stage improvised plays (as documented by the observers’ field notes) with adult company taking on the role of the audience (hence the high engagement scores for adults – see below). In U4, the field notes indicate that U4 appealed more to boys and that the older children’s play was more goal-oriented (building the city).

Decrease of adults’ engagement in U4 is likely related to the presence of seating for parents, who often consider these expositional segments as opportunities to rest and let the children play on their own (this is documented by the observers’ field notes). Interestingly, adults accompanying toddler–preschooler groups engaged more in U4 compared to those with older children. While observers noted that younger children were frequently left to “play” on the ground with parents seemingly indifferent to the activity’s purpose, the notes provide little insight into why adult engagement was higher in these groups. Based on occasional remarks, this may be linked to adults managing intra-group dynamics, such as conflicts between toddlers and preschoolers when playing with building blocks in U4.

Finally, facilitators typically do not engage except in U5, where they actively help the children with the hands-on activities. Facilitators’ engagement did not differ across age groups but was significantly associated with when during the week and in which slot the visit took place. During days with a high visit rate, facilitators’ engagement drops. It also tends to drop with the later slots. This is not surprising – fewer visitors enable facilitators to engage more closely. As the day progresses, fatigue seems to take its toll on the facilitators who are working the entire day shift. Apart from optimizing the workload, facilitators’ engagement can be enhanced by design adjustments. At U5, where they can provide books and help with worksheets, they know better what to do, and they are more familiar with this section’s content, facilitators were significantly more active than in the rest of the units. Incorporating into facilitators’ work provision of scripts, opening lines and sets of questions or other information for visitors might be useful. Additionally, facilitators should be involved directly in the development of scenarios for interaction [40].

The analysis revealed differences between units pertaining to the perception of labels, symbols and evocative questions. Symbols elicited most reactions in U1. As stated above, one of the purposes of the first exhibition hall is to evoke the themes of the ChM – the symbols representing the theme are displayed visibly on the wall facing the visitors when they progress to the second hall. The symbols are also projected on the interactive wall as part of the games as well as the accompanying graphics. Evocative questions failed to attract visitors’ attention in U1. This might be due to their location in the seating area: the texts are obstructed by the visitors sitting there. As for the labels, very few can be found in U1; hence, the low score for this unit. U2, on the other hand, is a label-rich area that resembles a “classical” museum setting with exhibits in displays with labels. The data did not allow for estimating the effect of age group on reactions to evocative questions, symbols and labels.

The overall lack of responsiveness to visual elements indicates that enhancing these aspects could provide a richer context for visitors to connect with the exhibits.

Inclusion of observer identity as a random effect allowed us to approximate the level of potential observer bias. While some interaction dimensions exhibited greater observer variance than others, overall variability was not substantial. The relatively high variance attributed to facilitator engagement appears to stem from its zero-inflated distribution, which likely amplifies perceived observer differences. When most observations cluster at the lowest score, even minor discrepancies in coding between observers disproportionately affect variance estimates.

## Conclusion

This study has presented the Children’s Museum as a part of the National Museum and its concept and design, which is unique and innovative in the context of Czech museum structures, as it is based on constructivism and interpretation of the exhibition is put in the hands of visitors. However, although this approach is well described in the literature, its implementation in ChM seems to be more challenging than expected. This may reflect the relatively high demands that this approach places on visitors’ motivation to engage actively with the exhibition. Our analysis suggests that many of the children currently visiting the ChM may be younger than the audience for whom the exhibition’s constructivist design was primarily intended. As a recommendation, it would be worthwhile to rethink the museum’s marketing strategy and focus more on school visits to attract older children. The findings suggest that while some units successfully encourage imaginative and collaborative interactions, others could benefit from more engaging exhibit designs to stimulate creativity. Even minor adjustments to unit design could potentially enhance the visitor experience. Besides the support of the facilitator, other improvements may include adding seats, provision of more content-related promotional materials with less emphasis on play, or reorganisation of the presentation of worksheets and books at U5.

The dynamics of the visit are likely influenced by the very first display U1, the interactive wall. It is questionable whether this scenario works well since the data suggest that this interactive display may not connect visitors thematically with the rest of the exhibition. It will be worthwhile to experiment with another entrance to the ChM through the U5, which is less distracting and should be understood as more ‘museum-like’. Museum visits in general are cognitively demanding and the play area at the end of the exhibition should be more appropriate as a kind of recharging facility. After playing at the beginning of the visit, it might be difficult for families to refocus on the subsequent exhibition content.

Our research draws on a large and diverse dataset, encompassing observations of more than 2000 individuals across five exhibition units and employing a mixed-method protocol combining quantitative and qualitative measures. This design provides a comprehensive perspective on visitor behaviour in a constructivist museum environment, offering insights into how exhibit design and family dynamics interact in practice. Our findings derive from a single constructivist museum setting and describe observable patterns of visitor behaviour rather than providing a general assessment of constructivist museum learning. Although visitor interaction and engagement represent only indirect indicators of learning processes, they contribute to ongoing discussions about the application of constructivist principles in museum settings and may inform strategies for creating exhibitions that balance playfulness with educational depth. In this respect, the study also contributes to research on the educational role of museums and other cultural institutions, consistent with the objectives of SDG 4 (Quality Education).

One of the main limitations of this research is the limited number of units observed. This is because the exhibition area is very large. This study works with the family as a unit, but it was observed that family members split up during their visits and later regroup, which could affect the results. Alternative methods, such as mental maps or tracking families through the whole space were also considered (cf. [41], for creative collaborative and participatory methods of children museum engagement analysis), but we chose to compare the particular units as a means more suitable to addressing our research questions. This research should be understood as a first probe into what really happens in the ChM. On the other hand, a substantial amount of qualitative data was gathered via observers’ field notes, field diaries, three collective reflections (debates) and also from interviews with the visitors. We plan to describe the quality of the different types of interaction and fill in the gaps that may remain in this study.
