# Supplementary material for: Too young to ask their own questions: Learning and visitor dynamics in a constructivist museum environment
Source: PLoS One. 2026 Aug 21;21(8):e0355543. doi: 10.1371/journal.pone.0355543 (PMC13496454; doi:10.1371/journal.pone.0355543)
Supplement: Supplementary file 1 [file pone.0355543.s003.pdf]

Response to Reviewers

Manuscript ID: PONE-D-25-27943R1
Title: *Too young to ask their own questions: learning and visitor dynamics in a constructivist museum environment*
Authors: Nina Wanča, Jakub Jehlička, Anna Hála

Dear Editors,

We thank the Academic Editor and the reviewers for their careful evaluation of our manuscript and for their constructive comments. Below we address the remaining issues raised in the decision letter.

Some of the subjective statements go beyond what is supported by the current evidence. Please adjust your claims and arguments, particularly in the "Discussion" and "Conclusion" sections.

We revised the "Discussion" and "Conclusion" sections to ensure that interpretive statements remain closely aligned with the observational nature of the data. In particular, we softened several claims regarding visitor behaviour, constructivist learning, and the practical implications of the findings. The corresponding revisions are highlighted throughout the marked-up manuscript.

In the second-to-last paragraph of the "Discussion" section, please clarify the theoretical, methodological, or practical limitations of the conclusions or the proposed model.

The manuscript already contained a dedicated limitations paragraph at the end of the "Conclusion". Rather than duplicating these points in the "Discussion", we expanded the preceding concluding paragraph by explicitly clarifying the theoretical scope of the conclusions while retaining the dedicated limitations section.

Original paragraph:

> "Our research draws on a large and diverse dataset, gathering observations of more than 2,000 individuals across five exhibition units and employing a mixed-method protocol combining quantitative and qualitative measures. This design provides a comprehensive perspective on visitor behavior in a constructivist museum environment, offering insights into how exhibit design and family dynamics interact in practice. These findings contribute to ongoing discussions about the translation of constructivist principles into museum settings and may inform strategies for creating exhibitions that balance playfulness with educational depth."

Revised passage:

> "Our research draws on a large and diverse dataset, encompassing observations of more than 2,000 individuals across five exhibition units and employing a mixed-method protocol combining quantitative and qualitative measures. This design provides a comprehensive perspective on visitor behavior in a constructivist museum environment, offering insights into how exhibit design and family dynamics interact in practice. Our findings derive from a single constructivist museum setting and describe observable patterns of visitor behaviour rather than providing a general assessment of constructivist museum learning. Although visitor interaction and engagement represent only indirect indicators of

> learning processes, they contribute to ongoing discussions about the application of constructivist principles in museum settings and may inform strategies for creating exhibitions that balance playfulness with educational depth. In this respect, the study also contributes to research on the educational role of museums and other cultural institutions consistent with the objectives of SDG 4 (Quality Education)."

The subsequent limitations paragraph (retained from the previous version) continues to address additional methodological limitations, including the limited number of observation points and the absence of individual-level analyses.

You may draw a connection to the SDGs at the end of the conclusion.

Following the reviewer's suggestion, we added a concluding sentence placing the study in the broader context of SDG 4 (Quality Education):

"In this respect, the study also contributes to research on the educational role of museums and other cultural institutions, consistent with the objectives of SDG 4 (Quality Education)."
