# Supplementary material for: Too young to ask their own questions: Learning and visitor dynamics in a constructivist museum environment
Source: PLoS One. 2026 Aug 21;21(8):e0355543. doi: 10.1371/journal.pone.0355543 (PMC13496454; doi:10.1371/journal.pone.0355543)
Supplement: Supplementary file 2 [file pone.0355543.s002.docx]

**Rebuttal Letter**

**Manuscript ID:** PONE-D-25-27943
**Title:** *Too young to ask their own questions: Visitor experience in the first year of the new Children's Museum*
**Authors:** Nina Wanča, Jakub Jehlička, Anna Doušová

Dear Editors,

Thank you for the opportunity to revise our manuscript and for the constructive feedback provided by the reviewers. We have considered all of the remarks and revised our manuscript accordingly. The revised version (both marked-up and clean) is attached. Below, we provide a point-by-point response to each comment.

**Reviewer #1**:

*The study of the Children's Museum offers a compelling exploration of an innovative initiative within the Czech museum landscape, particularly due to its integration within the National Museum and its alignment with constructivist educational principles. The museum’s emphasis on visitor-led interpretation and interactive engagement is commendable, however, the manuscript would benefit from a more robust theoretical and methodological foundation to enhance its academic contribution.

The introduction currently lacks sufficient engagement with relevant theoretical frameworks, particularly those related to constructivism in museum education. A more comprehensive literature review, including both conceptual discussions and empirical studies of constructivist approaches in museum settings, would provide a stronger foundation. Additionally, comparative insights from similar children’s museums or interactive exhibitions internationally would help contextualize the distinctiveness of the museum.*

**REPLY**: We have addressed this issue by providing additional theoretical grounding throughout the text, not only the Introduction. We have provided comparative perspective regarding children’s museums world-wide and relevant studies on observational research in children’s museums. We enhanced the theoretical grounding of our paper by supporting it with references to relevant works on constructivist pedagogy, visitor studies, and observational research (the reference list has been expanded from 19 to 41 items).

*The section titled The Children's Museum primarily describes the study setting and would be more appropriately situated within the Methods section. This repositioning would clarify the structure and improve the logical flow of the manuscript. Furthermore, the methodology section requires greater transparency. While the involvement of an ethnologist is noted, the theoretical lens guiding the observations remains unspecified. Clarifying the ethnographic tradition, observation protocols, and analytical procedures would significantly strengthen the study’s methodological rigor.*

**REPLY**: The section “The Children’s Museum” has been incorporated into the “Methods” section as a subsection titled “Study setting”, and the original beginning of “Methods” section has been turned into a subsection of the Methods section titled “Objectives”.

The objectives section has been supplemented with additional literature review on observational research in the context of children’s museums to provide better anchoring of our study’s theoretical and methodological framing.

The “Methods” section now includes explicit details on the theoretical lens and ethnographic tradition guiding the observations – the subsection “Observation procedure” has been amended in this vein and several references (on ethnographic methodological background) were added.

*The introduction of the three principles of interaction—Learning, Playing, and Creating—appears to stem from pilot observations, yet the process of their development is insufficiently detailed. These categories would benefit from clearer operationalization and grounding in existing literature on informal learning and museum pedagogy.*

**REPLY**: The process of the development of the interaction categories has been clarified in the “Observed phenomena” subsection. We have addressed the particular steps taken during the development process, supported by references to works relevant for theoretical grounding of the elaborated concepts. We have addressed the problem of operationalization by providing simplified definitions of the observed behaviors.

*A recurring limitation throughout the manuscript is the lack of theoretical and comparative context. The study would be enriched by positioning its findings within broader discourses on museum learning, visitor studies, and constructivist pedagogy. Moreover, the absence of comparative analysis with similar initiatives limits the generalizability and broader relevance of the findings.*

**REPLY**: We have revised all sections of the paper in order to position it more explicitly within the theoretical context: we added a number of references to relevant parts to support our theoretical grounding as well as findings. The list of references was expanded from 19 to 41 items, most of them regarding constructivist pedagogy, child visitor behaviour and engagement and methodology of observational research in the context of children exhibitions. We anchored our study in the context of similar studies by providing a more coherent review of relevant recent studies focusing on observational research in children’s museum settings.

*Finally, the manuscript would be strengthened by a more explicit articulation of its contribution to the field. Clarifying the new insights it offers into the design and evaluation of children’s museums, and how these findings might inform future research or practice, would help situate the study within a broader academic and practical discourse.*

**REPLY**: Contributions and implications of our study more explicitly formulated in the Conclusion section, esp. in lines 746–752.

**Reviewer #2:**

*This manuscript examines visitor engagement and learning dynamics in a newly established Children’s Museum in Prague, designed around constructivist pedagogical principles. Using 720 structured observations of over 2,000 visitors, the authors analyze how different age cohorts, accompanying adults, and facilitators interact with exhibits. The study identifies a significant mismatch between the intended audience (ages 6–10) and the actual predominant visitors (preschoolers), raising questions about the accessibility of the museum’s constructivist design. The paper is well-structured, clearly written, and contributes valuable empirical evidence to the field of museum education.*

*Strengths

A large dataset of systematically collected observational evidence supports the analyses.

The study addresses a relevant and underexplored question: the alignment of constructivist museum design with actual visitor demographics.

The open sharing of data and R scripts via OSF enhances transparency and reproducibility.

The discussion effectively links findings to broader debates in constructivist pedagogy and museum learning.

Weaknesses and Limitations

Overgeneralization of findings – The conclusion that preschool-aged children are “too young” for constructivist approaches may be overstated. Although qualitative field notes and interviews were collected, these are not adequately integrated into the main analysis to substantiate claims.

Analytical rigor – While appropriate non-parametric tests are used, the analysis is limited to group comparisons. Effect sizes, confidence intervals, and multivariate analyses would strengthen the robustness of the findings.

Observer reliability – The manuscript does not report inter-rater reliability or calibration among the 19 observers. This omission raises concerns about measurement consistency across such a large team.*

*Sampling scope – The study focuses on only five observation units within a large and complex exhibition. Families often split and regroup during visits, which may limit the representativeness of the data.

Repeated measures – The issue of potentially duplicated observations (approximately 19% of visitors) is acknowledged but not fully addressed analytically, introducing a possible bias.

Facilitator role – The paper emphasizes facilitator passivity but does not sufficiently explore underlying reasons. More triangulation with qualitative data could provide insight into this dynamic.

Presentation – While generally well written, sections of the manuscript are repetitive, and results could be streamlined for greater clarity and concision.*

**Recommendations**

*The manuscript provides an important empirical contribution to the field of museum learning and constructivist pedagogy. However, before publication, I recommend that the authors:

Provide additional evidence from field notes or interviews to support claims about preschoolers’ inability to engage constructively.*

**REPLY**: Our current paper focuses primarily on the quantitative aspects of the observations, and we have emphasized this focus at several points in the manuscript. While we agree that qualitative insights from field notes and interviews would provide valuable context, we believe that a thorough qualitative analysis deserves a separate study to do justice to its depth and complexity. For this reason, we have limited the use of field notes in the present paper to brief illustrative examples only. We plan to address these qualitative dimensions in detail in a subsequent publication.

*Report inter-rater reliability for observational data.*

**REPLY**: While we did not report inter-rater reliability statistics in the manuscript, we took several steps to ensure consistency across the 19 observers. Importantly, the observations were independent, with each observer stationed at a single unit during a given time slot. This means that no overlapping observations of the same visitor group at the same unit occurred, which limits the applicability of traditional inter-rater reliability metrics, such as Cohen’s kappa. We mentioned regular calibration meetings on lines 249–250: “To ensure the consistency of the observations across units and individual observers, periodical feedback sessions with the entire team were held.”

However, inter-coder variability is now thoroughly addressed by including observers as random effects in the mixed-effects regression models. This way, we were able to assess the magnitude of the effect of possible observer idiosyncrasies over specific measures. Treating variability as a parameter within the multivariate analysis is more robust than relying on standard reliability coefficients, as it accounts for differences directly in the modelling framework.

*Include effect sizes and, where possible, multivariate analyses to strengthen the statistical rigor.*

**REPLY**: We have substantially revised the analytical approach. The original interaction and engagement analyses have been replaced with mixed-effects ordinal regression models, which allow for a more robust and coherent treatment of the data and account for clustering by observer. Importantly, the substantive conclusions remain consistent with those reported in the original analyses.

These models incorporate multiple predictors and interaction terms, thereby providing a multivariate perspective on visitor behavior. The new models confirm the same key patterns while offering more precise estimates and effect sizes (odds ratios with 95% confidence intervals).

To maintain readability, we kept the structure of the Results section concise and avoided presenting the full tabular output in the main text, as these tables are very large. Instead, we provide complete model summaries and detailed estimates (including odds ratios and confidence intervals) in the Supplementary information. In the text, we report significant effects (odds ratios and 95% confidence intervals).

For all remaining statistical tests, we now report effect sizes.

*Clarify how potential duplicate observations were handled analytically.*

**REPLY**: There are no duplicate observations in our data. In our study, by *repeated* observations we mean visitor groups that were observed more than once during their visit, however, they were never observed by two observers at the same time. These cases occurred only when the same visitor group was observed at different units during the same visit. For analyses of group composition, we addressed this by using a reduced dataset that excluded potentially repeated cases (so that one visitor would not be counted twice). For analyses of interaction and engagement, however, each observation was treated as unit-specific, reflecting the distinct affordances and design features of each exhibition segment. Since our aim was not to track individual-level change across units, we are confident that this approach is appropriate and does not introduce bias as each observation represents a unique instance of a particular visitor group at a specific place and time.

We clarify this in the manuscript on lines 359–362.

*Streamline presentation by reducing redundancy in the results and discussion sections.*

**REPLY**: We have dealt with this issue mainly by dividing Discussion and Conclusion into separate sections. “Discussion” now focuses on interpreting the results, while “Conclusion” is dedicated to generalizations, implications, limitations, and future directions. We believe this separation makes the argument clearer and reduces redundancy between sections.

**Reviewer #3:**

*1.The author does not articulate how its findings extend, challenge, or refine constructivism within the specific context of museum learning.*

**REPLY**: Addressed above (see notes on review #1)

*2.To insufficiently situates the Czech museum environment within the broader European or global landscape of children’s museums.*

**REPLY**: We have added a paragraph with more context on children’s museums from a global perspective.

*3.Potential external determinants of visitor composition—such as socioeconomic background, ticket pricing, accessibility, or educational disparities—are not examined. These could have provided valuable insight into why younger, not older, children dominate visitation.*

**REPLY**: We agree that this kind of information would provide valuable insight. However, we carried out non-participant observation, and this method did not allow us to collect demographic and other visitor-related data.

*4.Observations were limited to five exhibition units, excluding other spaces that might provide complementary insights.*

**REPLY**: We acknowledge that focusing on five observation units may restrict the representativeness of the findings. These units were selected deliberately to capture the diversity of interaction principles and thematic content across the exhibition (see Table 2). While this approach allowed for systematic comparison of distinct affordances, we recognize that other spaces could provide complementary insights. We have clarified this limitation in the Discussion and Conclusion sections and suggested that future research should include additional units or employ whole-exhibition tracking methods to provide a more comprehensive view.
