# Supplementary material for: Too young to ask their own questions: Learning and visitor dynamics in a constructivist museum environment
Source: PLoS One. 2026 Aug 21;21(8):e0355543. doi: 10.1371/journal.pone.0355543 (PMC13496454; doi:10.1371/journal.pone.0355543)
Supplement: Peer review history

# Peer review: Decision Letter 0

**DOI:** 10.1371/journal.pone.0355543.r001

**Authors:** Weicong Li

13 Oct 2025

Dear Dr. Jehlicka,

Thank you for submitting your manuscript to PLOS ONE. After careful consideration, we feel that it has merit but does not fully meet PLOS ONE’s publication criteria as it currently stands. Therefore, we invite you to submit a revised version of the manuscript that addresses the points raised during the review process.

Please submit your revised manuscript by Nov 27 2025 11:59PM. If you will need more time than this to complete your revisions, please reply to this message or contact the journal office at plosone@plos.org. When you're ready to submit your revision, log on to https://www.editorialmanager.com/pone/ and select the 'Submissions Needing Revision' folder to locate your manuscript file.

- A rebuttal letter that responds to each point raised by the academic editor and reviewer(s). You should upload this letter as a separate file labeled 'Response to Reviewers'.
- A marked-up copy of your manuscript that highlights changes made to the original version. You should upload this as a separate file labeled 'Revised Manuscript with Track Changes'.
- An unmarked version of your revised paper without tracked changes. You should upload this as a separate file labeled 'Manuscript'.

If you would like to make changes to your financial disclosure, please include your updated statement in your cover letter. Guidelines for resubmitting your figure files are available below the reviewer comments at the end of this letter.

If applicable, we recommend that you deposit your laboratory protocols in protocols.io to enhance the reproducibility of your results. Protocols.io assigns your protocol its own identifier (DOI) so that it can be cited independently in the future. For instructions see: https://journals.plos.org/plosone/s/submission-guidelines#loc-laboratory-protocols. Additionally, PLOS ONE offers an option for publishing peer-reviewed Lab Protocol articles, which describe protocols hosted on protocols.io. Read more information on sharing protocols at https://plos.org/protocols?utm_medium=editorial-email&utm_source=authorletters&utm_campaign=protocols.

We look forward to receiving your revised manuscript.

Kind regards,

Weicong Li, P.hD

Academic Editor

PLOS ONE

Journal requirements:

When submitting your revision, we need you to address these additional requirements.

1. Please ensure that your manuscript meets PLOS ONE's style requirements, including those for file naming. The PLOS ONE style templates can be found at

https://journals.plos.org/plosone/s/file?id=wjVg/PLOSOne_formatting_sample_main_body.pdf and

https://journals.plos.org/plosone/s/file?id=ba62/PLOSOne_formatting_sample_title_authors_affiliations.pdf

2. Thank you for stating the following financial disclosure:

“Charles University Research Centre Program No. UNCE/24/SSH/026

Ministry of Culture of the Czech Republic (DKRVO 2024–2028/26.I.a, National Museum).”

Please state what role the funders took in the study.  If the funders had no role, please state: "The funders had no role in study design, data collection and analysis, decision to publish, or preparation of the manuscript."

If this statement is not correct you must amend it as needed.

Please include this amended Role of Funder statement in your cover letter; we will change the online submission form on your behalf.

3. Please ensure that you include a title page within your main document. You should list all authors and all affiliations as per our author instructions and clearly indicate the corresponding author.

4. Please include captions for your Supporting Information files at the end of your manuscript, and update any in-text citations to match accordingly. Please see our Supporting Information guidelines for more information: http://journals.plos.org/plosone/s/supporting-information.

If the reviewer comments include a recommendation to cite specific previously published works, please review and evaluate these publications to determine whether they are relevant and should be cited. There is no requirement to cite these works unless the editor has indicated otherwise.

[Note: HTML markup is below. Please do not edit.]

Reviewers' comments:

Reviewer's Responses to Questions

**Comments to the Author**

1. Is the manuscript technically sound, and do the data support the conclusions?

Reviewer #1: Yes

Reviewer #2: Yes

Reviewer #3: Partly

**********

2. Has the statistical analysis been performed appropriately and rigorously?

Reviewer #1: Yes

Reviewer #2: Yes

Reviewer #3: N/A

**********

3. Have the authors made all data underlying the findings in their manuscript fully available?

The PLOS Data policy

Reviewer #1: Yes

Reviewer #2: Yes

Reviewer #3: Yes

**********

4. Is the manuscript presented in an intelligible fashion and written in standard English?

Reviewer #1: Yes

Reviewer #2: Yes

Reviewer #3: Yes

**********

Reviewer #1: The study of the Children's Museum offers a compelling exploration of an innovative initiative within the Czech museum landscape, particularly due to its integration within the National Museum and its alignment with constructivist educational principles. The museum’s emphasis on visitor-led interpretation and interactive engagement is commendable, however, the manuscript would benefit from a more robust theoretical and methodological foundation to enhance its academic contribution.

The introduction currently lacks sufficient engagement with relevant theoretical frameworks, particularly those related to constructivism in museum education. A more comprehensive literature review, including both conceptual discussions and empirical studies of constructivist approaches in museum settings, would provide a stronger foundation. Additionally, comparative insights from similar children’s museums or interactive exhibitions internationally would help contextualize the distinctiveness of the museum.

The section titled The Children's Museum primarily describes the study setting and would be more appropriately situated within the Methods section. This repositioning would clarify the structure and improve the logical flow of the manuscript. Furthermore, the methodology section requires greater transparency. While the involvement of an ethnologist is noted, the theoretical lens guiding the observations remains unspecified. Clarifying the ethnographic tradition, observation protocols, and analytical procedures would significantly strengthen the study’s methodological rigor.

The introduction of the three principles of interaction—Learning, Playing, and Creating—appears to stem from pilot observations, yet the process of their development is insufficiently detailed. These categories would benefit from clearer operationalization and grounding in existing literature on informal learning and museum pedagogy.

A recurring limitation throughout the manuscript is the lack of theoretical and comparative context. The study would be enriched by positioning its findings within broader discourses on museum learning, visitor studies, and constructivist pedagogy. Moreover, the absence of comparative analysis with similar initiatives limits the generalizability and broader relevance of the findings.

Finally, the manuscript would be strengthened by a more explicit articulation of its contribution to the field. Clarifying the new insights it offers into the design and evaluation of children’s museums, and how these findings might inform future research or practice, would help situate the study within a broader academic and practical discourse.

Reviewer #2: This manuscript examines visitor engagement and learning dynamics in a newly established Children’s Museum in Prague, designed around constructivist pedagogical principles. Using 720 structured observations of over 2,000 visitors, the authors analyze how different age cohorts, accompanying adults, and facilitators interact with exhibits. The study identifies a significant mismatch between the intended audience (ages 6–10) and the actual predominant visitors (preschoolers), raising questions about the accessibility of the museum’s constructivist design. The paper is well-structured, clearly written, and contributes valuable empirical evidence to the field of museum education.

Strengths

A large dataset of systematically collected observational evidence supports the analyses.

The study addresses a relevant and underexplored question: the alignment of constructivist museum design with actual visitor demographics.

The open sharing of data and R scripts via OSF enhances transparency and reproducibility.

The discussion effectively links findings to broader debates in constructivist pedagogy and museum learning.

Weaknesses and Limitations

Overgeneralization of findings – The conclusion that preschool-aged children are “too young” for constructivist approaches may be overstated. Although qualitative field notes and interviews were collected, these are not adequately integrated into the main analysis to substantiate claims.

Analytical rigor – While appropriate non-parametric tests are used, the analysis is limited to group comparisons. Effect sizes, confidence intervals, and multivariate analyses would strengthen the robustness of the findings.

Observer reliability – The manuscript does not report inter-rater reliability or calibration among the 19 observers. This omission raises concerns about measurement consistency across such a large team.

Sampling scope – The study focuses on only five observation units within a large and complex exhibition. Families often split and regroup during visits, which may limit the representativeness of the data.

Repeated measures – The issue of potentially duplicated observations (approximately 19% of visitors) is acknowledged but not fully addressed analytically, introducing a possible bias.

Facilitator role – The paper emphasizes facilitator passivity but does not sufficiently explore underlying reasons. More triangulation with qualitative data could provide insight into this dynamic.

Presentation – While generally well written, sections of the manuscript are repetitive, and results could be streamlined for greater clarity and concision.

Recommendations

The manuscript provides an important empirical contribution to the field of museum learning and constructivist pedagogy. However, before publication, I recommend that the authors:

Provide additional evidence from field notes or interviews to support claims about preschoolers’ inability to engage constructively.

Report inter-rater reliability for observational data.

Include effect sizes and, where possible, multivariate analyses to strengthen the statistical rigor.

Clarify how potential duplicate observations were handled analytically.

Streamline presentation by reducing redundancy in the results and discussion sections.

Reviewer #3: 1.The author does not articulate how its findings extend, challenge, or refine constructivism within the specific context of museum learning.

2.To insufficiently situates the Czech museum environment within the broader European or global landscape of children’s museums.

3.Potential external determinants of visitor composition—such as socioeconomic background, ticket pricing, accessibility, or educational disparities—are not examined. These could have provided valuable insight into why younger, not older, children dominate visitation.

4.Observations were limited to five exhibition units, excluding other spaces that might provide complementary insights.

**********

what does this mean?). If published, this will include your full peer review and any attached files.

If you choose “no”, your identity will remain anonymous but your review may still be made public.

**Do you want your identity to be public for this peer review?**  For information about this choice, including consent withdrawal, please see our Privacy Policy

Reviewer #1: No

Reviewer #2: No

Reviewer #3: No

**********

[NOTE: If reviewer comments were submitted as an attachment file, they will be attached to this email and accessible via the submission site. Please log into your account, locate the manuscript record, and check for the action link "View Attachments". If this link does not appear, there are no attachment files.]

While revising your submission, please upload your figure files to the Preflight Analysis and Conversion Engine (PACE) digital diagnostic tool, https://pacev2.apexcovantage.com/. PACE helps ensure that figures meet PLOS requirements. To use PACE, you must first register as a user. Registration is free. Then, login and navigate to the UPLOAD tab, where you will find detailed instructions on how to use the tool. If you encounter any issues or have any questions when using PACE, please email PLOS at figures@plos.org. Please note that Supporting Information files do not need this step.

# Author comment: Author response to Decision Letter 1

**DOI:** 10.1371/journal.pone.0355543.r002

26 Nov 2025

Reviewer #1:

The study of the Children's Museum offers a compelling exploration of an innovative initiative within the Czech museum landscape, particularly due to its integration within the National Museum and its alignment with constructivist educational principles. The museum’s emphasis on visitor-led interpretation and interactive engagement is commendable, however, the manuscript would benefit from a more robust theoretical and methodological foundation to enhance its academic contribution.

The introduction currently lacks sufficient engagement with relevant theoretical frameworks, particularly those related to constructivism in museum education. A more comprehensive literature review, including both conceptual discussions and empirical studies of constructivist approaches in museum settings, would provide a stronger foundation. Additionally, comparative insights from similar children’s museums or interactive exhibitions internationally would help contextualize the distinctiveness of the museum.

#############################################################

REPLY: We have addressed this issue by providing additional theoretical grounding throughout the text, not only the Introduction. We have provided comparative perspective regarding children’s museums world-wide and relevant studies on observational research in children’s museums. We enhanced the theoretical grounding of our paper by supporting it with references to relevant works on constructivist pedagogy, visitor studies, and observational research (the reference list has been

expanded from 19 to 41 items).

#############################################################

The section titled The Children's Museum primarily describes the study setting and would be more appropriately situated within the Methods section. This repositioning would clarify the structure and improve the logical flow of the manuscript. Furthermore, the methodology section requires greater transparency. While the involvement of an ethnologist is noted, the theoretical lens guiding the observations remains unspecified. Clarifying the ethnographic tradition, observation protocols, and

analytical procedures would significantly strengthen the study’s methodological rigor.

#############################################################

REPLY: The section “The Children’s Museum” has been incorporated into the “Methods” section as a subsection titled “Study setting”, and the original beginning of “Methods” section has been turned into a subsection of the Methods section titled “Objectives”.

The objectives section has been supplemented with additional literature review on observational research in the context of children’s museums to provide better anchoring of our study’s theoretical and methodological framing.

The “Methods” section now includes explicit details on the theoretical lens and ethnographic tradition guiding the observations – the subsection “Observation procedure” has been amended in this vein and several references (on ethnographic methodological background) were added.

#############################################################

The introduction of the three principles of interaction—Learning, Playing, and Creating—appears to stem from pilot observations, yet the process of their development is insufficiently detailed. These categories would benefit from clearer operationalization and grounding in existing literature on informal learning and museum pedagogy.

#############################################################

REPLY: The process of the development of the interaction categories has been clarified in the “Observed phenomena” subsection. We have addressed the particular steps taken during the development process, supported by references to works relevant for theoretical grounding of the elaborated concepts. We have addressed the problem of operationalization by providing simplified definitions of the observed behaviors.

#############################################################

A recurring limitation throughout the manuscript is the lack of theoretical and comparative context. The study would be enriched by positioning its findings within broader discourses on museum learning, visitor studies, and constructivist pedagogy. Moreover, the absence of comparative analysis with similar initiatives limits the generalizability and broader relevance of the findings.

#############################################################

REPLY: We have revised all sections of the paper in order to position it more explicitly within the theoretical context: we added a number of references to relevant parts to support our theoretical grounding as well as findings. The list of references was expanded from 19 to 41 items, most of them regarding constructivist pedagogy, child visitor behaviour and engagement and methodology of observational research in the context of children exhibitions. We anchored our study in the context of similar studies by providing a more coherent review of relevant recent studies focusing on observational research in children’s museum settings.

#############################################################

Finally, the manuscript would be strengthened by a more explicit articulation of its contribution to the field. Clarifying the new insights it offers into the design and evaluation of children’s museums, and how these findings might inform future research or practice, would help situate the study within a broader academic and practical discourse.

#############################################################

REPLY: Contributions and implications of our study have been more explicitly formulated in the Conclusion

section, esp. in lines 746–752.

#############################################################

Reviewer #2:

This manuscript examines visitor engagement and learning dynamics in a newly established

Children’s Museum in Prague, designed around constructivist pedagogical principles. Using 720 structured observations of over 2,000 visitors, the authors analyze how different age cohorts, accompanying adults, and facilitators interact with exhibits. The study identifies a significant mismatch between the intended audience (ages 6–10) and the actual predominant visitors (preschoolers), raising questions about the accessibility of the museum’s constructivist design. The paper is well-structured, clearly written, and contributes valuable empirical evidence to the field of museum education.

Strengths

A large dataset of systematically collected observational evidence supports the analyses.

The study addresses a relevant and underexplored question: the alignment of constructivist museum design with actual visitor demographics.

The open sharing of data and R scripts via OSF enhances transparency and reproducibility.

The discussion effectively links findings to broader debates in constructivist pedagogy and museum learning.

Weaknesses and Limitations

Overgeneralization of findings – The conclusion that preschool-aged children are “too young” for constructivist approaches may be overstated. Although qualitative field notes and interviews were collected, these are not adequately integrated into the main analysis to substantiate claims.

Analytical rigor – While appropriate non-parametric tests are used, the analysis is limited to group comparisons. Effect sizes, confidence intervals, and multivariate analyses would strengthen the robustness of the findings.

Observer reliability – The manuscript does not report inter-rater reliability or calibration among the 19 observers. This omission raises concerns about measurement consistency across such a large team.

Sampling scope – The study focuses on only five observation units within a large and complex exhibition. Families often split and regroup during visits, which may limit the representativeness of the data.

Repeated measures – The issue of potentially duplicated observations (approximately 19% of visitors) is acknowledged but not fully addressed analytically, introducing a possible bias.

Facilitator role – The paper emphasizes facilitator passivity but does not sufficiently explore

underlying reasons. More triangulation with qualitative data could provide insight into this dynamic.

Presentation – While generally well written, sections of the manuscript are repetitive, and results could be streamlined for greater clarity and concision.

Recommendations

The manuscript provides an important empirical contribution to the field of museum learning and constructivist pedagogy. However, before publication, I recommend that the authors:

Provide additional evidence from field notes or interviews to support claims about preschoolers’ inability to engage constructively.

#############################################################

REPLY: Our current paper focuses primarily on the quantitative aspects of the observations, and we have emphasized this focus at several points in the manuscript. While we agree that qualitative insights from field notes and interviews would provide valuable context, we believe that a thorough qualitative analysis deserves a separate study to do justice to its depth and complexity. For this reason, we have limited the use of field notes in the present paper to brief illustrative examples only. We plan to address these qualitative dimensions in detail in a subsequent publication.

#############################################################

Report inter-rater reliability for observational data.

#############################################################

REPLY: While we did not report inter-rater reliability statistics in the manuscript, we took several steps to ensure consistency across the 19 observers. Importantly, the observations were independent, with each observer stationed at a single unit during a given time slot. This means that no overlapping observations of the same visitor group at the same unit occurred, which limits the applicability of traditional inter-rater reliability metrics, such as Cohen’s kappa. We mentioned regular calibration meetings on lines 249–250: “To ensure the consistency of the observations across units and individual observers, periodical feedback sessions with the entire team were held.”

However, inter-coder variability is now thoroughly addressed by including observers as random effects in the mixed-effects regression models. This way, we were able to assess the magnitude of the effect of possible observer idiosyncrasies over specific measures. Treating variability as a parameter within the multivariate analysis is more robust than relying on standard reliability coefficients, as it accounts for differences directly in the modelling framework.

#############################################################

Include effect sizes and, where possible, multivariate analyses to strengthen the statistical rigor.

#############################################################

REPLY: We have substantially revised the analytical approach. The original interaction and engagement analyses have been replaced with mixed-effects ordinal regression models, which allow for a more robust and coherent treatment of the data and account for clustering by observer. Importantly, the substantive conclusions remain consistent with those reported in the original analyses. These models incorporate multiple predictors and interaction terms, thereby providing a multivariate perspective on visitor behavior. The new models confirm the same key patterns while offering more precise estimates and effect sizes (odds ratios with 95% confidence intervals). To maintain readability, we kept the structure of the Results section concise and avoided presenting the full tabular output in the main text, as these tables are very large. Instead, we provide complete model summaries and detailed estimates (including odds ratios and confidence intervals) in the Supplementary information. In the text, we report significant effects (odds ratios and 95% confidence intervals).

For all remaining statistical tests, we now report effect sizes.

#############################################################

Clarify how potential duplicate observations were handled analytically.

#############################################################

REPLY: There are no duplicate observations in our data. In our study, by repeated observations we mean visitor groups that were observed more than once during their visit, however, they were never observed by two observers at the same time. These cases occurred only when the same visitor group was observed at different units during the same visit. For analyses of group composition, we addressed this by using a reduced dataset that excluded potentially repeated cases (so that one visitor would not be counted twice). For analyses of interaction and engagement, however, each observation was treated as unit-specific, reflecting the distinct affordances and design features of each exhibition segment. Since our aim was not to track individual-level change across units, we are confident that this approach is appropriate and does not introduce bias as each observation represents a unique instance of a particular visitor group at a specific place and time.

We clarify this in the manuscript on lines 359–362.

#############################################################

Streamline presentation by reducing redundancy in the results and discussion sections.

#############################################################

REPLY: We have dealt with this issue mainly by dividing Discussion and Conclusion into separate sections. “Discussion” now focuses on interpreting the results, while “Conclusion” is dedicated to generalizations, implications, limitations, and future directions. We believe this separation makes the argument clearer and reduces redundancy between sections.

Reviewer #3:

1.The author does not articulate how its findings extend, challenge, or refine constructivism within the specific context of museum learning.

#############################################################

REPLY: Addressed above (see notes on review #1)

#############################################################

2.To insufficiently situates the Czech museum environment within the broader European or global landscape of children’s museums.

#############################################################

REPLY: We have added a paragraph with more context on children’s museums from a global perspective.

#############################################################

3.Potential external determinants of visitor composition—such as socioeconomic background, ticket pricing, accessibility, or educational disparities—are not examined. These could have provided valuable insight into why younger, not older, children dominate visitation.

#############################################################

REPLY: We agree that this kind of information would provide valuable insight. However, we carried out non-participant observation, and this method did not allow us to collect demographic and other visitor-related data.

#############################################################

4.Observations were limited to five exhibition units, excluding other spaces that might provide complementary insights.

#############################################################

REPLY: We acknowledge that focusing on five observation units may restrict the representativeness of the findings. These units were selected deliberately to capture the diversity of interaction principles and thematic content across the exhibition (see Table 2). While this approach allowed for systematic comparison of distinct affordances, we recognize that other spaces could provide complementary insights. We have clarified this limitation in the Discussion and Conclusion sections and suggested that future research should include additional units or employ whole-exhibition tracking methods to provide a more comprehensive view.

#############################################################

Editor comments:

1.Please ensure that your manuscript meets PLOS ONE's style requirements, including those for file naming.

### REPLY: Manuscript has been edited in accordance to the style requirements. Fi

Attachment Submitted filename: rebuttal_letter_PONE-D-25-27943.docx

# Peer review: Decision Letter 1

**DOI:** 10.1371/journal.pone.0355543.r003

**Authors:** Weicong Li

12 Jul 2026

Dear Dr. Jehlicka,

Thank you for submitting your manuscript to PLOS ONE. After careful consideration, we feel that it has merit but does not fully meet PLOS ONE’s publication criteria as it currently stands. Therefore, we invite you to submit a revised version of the manuscript that addresses the points raised during the review process.

Please submit your revised manuscript by Aug 26 2026 11:59PM. If you will need more time than this to complete your revisions, please reply to this message or contact the journal office at plosone@plos.org. When you're ready to submit your revision, log on to https://www.editorialmanager.com/pone/ and select the 'Submissions Needing Revision' folder to locate your manuscript file.

- A letter that responds to each point raised by the academic editor and reviewer(s). You should upload this letter as a separate file labeled 'Response to Reviewers'.
- A marked-up copy of your manuscript that highlights changes made to the original version. You should upload this as a separate file labeled 'Revised Manuscript with Track Changes'.
- An unmarked version of your revised paper without tracked changes. You should upload this as a separate file labeled 'Manuscript'.

If you would like to make changes to your financial disclosure, please include your updated statement in your cover letter. Guidelines for resubmitting your figure files are available below the reviewer comments at the end of this letter.

If applicable, we recommend that you deposit your laboratory protocols in protocols.io to enhance the reproducibility of your results. Protocols.io assigns your protocol its own identifier (DOI) so that it can be cited independently in the future. For instructions see: https://journals.plos.org/plosone/s/submission-guidelines#loc-laboratory-protocols. Additionally, PLOS ONE offers an option for publishing peer-reviewed Lab Protocol articles, which describe protocols hosted on protocols.io. Read more information on sharing protocols at https://plos.org/protocols?utm_medium=editorial-email&utm_source=authorletters&utm_campaign=protocols.

As the corresponding author, your ORCID iD is verified in the submission system and will appear in the published article. PLOS supports the use of ORCID, and we encourage all coauthors to register for an ORCID iD and use it as well. Please encourage your coauthors to verify their ORCID iD within the submission system before final acceptance, as unverified ORCID iDs will not appear in the published article. *Only*  the individual author can complete the verification step; PLOS staff *cannot*  verify ORCID iDs on behalf of authors.

We look forward to receiving your revised manuscript.

Kind regards,

Weicong Li, P.hD

Academic Editor

PLOS One

Journal Requirements:

If the reviewer comments include a recommendation to cite specific previously published works, please review and evaluate these publications to determine whether they are relevant and should be cited. There is no requirement to cite these works unless the editor has indicated otherwise.

Please review your reference list to ensure that it is complete and correct. If you have cited papers that have been retracted, please include the rationale for doing so in the manuscript text, or remove these references and replace them with relevant current references. Any changes to the reference list should be mentioned in the rebuttal letter that accompanies your revised manuscript. If you need to cite a retracted article, indicate the article’s retracted status in the References list and also include a citation and full reference for the retraction notice.

Additional Editor Comments (if provided):

find my comments in reviewer 3.

[Note: HTML markup is below. Please do not edit.]

Reviewers' comments:

Reviewer's Responses to Questions

**Comments to the Author**

Reviewer #2: All comments have been addressed

Reviewer #3: All comments have been addressed

Reviewer #4: All comments have been addressed

**********

2. Is the manuscript technically sound, and do the data support the conclusions?

Reviewer #2: Yes

Reviewer #3: Partly

Reviewer #4: Yes

**********

3. Has the statistical analysis been performed appropriately and rigorously?

Reviewer #2: Yes

Reviewer #3: N/A

Reviewer #4: Yes

**********

4. Have the authors made all data underlying the findings in their manuscript fully available?

The PLOS Data policy

Reviewer #2: Yes

Reviewer #3: Yes

Reviewer #4: Yes

**********

5. Is the manuscript presented in an intelligible fashion and written in standard English?

Reviewer #2: Yes

Reviewer #3: Yes

Reviewer #4: Yes

**********

Reviewer #2: Dear Author congratulations for reaching at this stage. Thank you you have addressed all of my comments well.

Reviewer #3: 1. Some of the subjective statements go beyond what is supported by the current evidence. Please adjust your claims and arguments, particularly in the “Discussion” and “Conclusion” sections.

2. You may draw a connection to the SDGs at the end of the conclusion.

3. In the second-to-last paragraph of the “Discussion” section, please clarify the theoretical, methodological, or practical limitations of the conclusions or the proposed model.

Reviewer #4: The paper is well-structured, clearly written, and contributes valuable empirical evidence to the field of museum education. The authors clarifying the ethnographic tradition, observation protocols, and analytical procedures had significantly strengthen the study’s methodological rigor & sound evidence.

**********

what does this mean?). If published, this will include your full peer review and any attached files.

If you choose “no”, your identity will remain anonymous but your review may still be made public.

**Do you want your identity to be public for this peer review?**  For information about this choice, including consent withdrawal, please see our Privacy Policy

Reviewer #2: No

Reviewer #3: No

Reviewer #4: **Yes:** MOHAMAD IBRANI SHAHRIMIN ADAM ASSIM

**********

[NOTE: If reviewer comments were submitted as an attachment file, they will be attached to this email and accessible via the submission site. Please log into your account, locate the manuscript record, and check for the action link "View Attachments". If this link does not appear, there are no attachment files.]

To ensure your figures meet our technical requirements, please review our figure guidelines: https://journals.plos.org/plosone/s/figures

You may also use PLOS’s free figure tool, NAAS, to help you prepare publication quality figures: https://journals.plos.org/plosone/s/figures#loc-tools-for-figure-preparation.

NAAS will assess whether your figures meet our technical requirements by comparing each figure against our figure specifications.

# Author comment: Author response to Decision Letter 2

**DOI:** 10.1371/journal.pone.0355543.r004

22 Jul 2026

Dear Editors,

We thank the Academic Editor and the reviewers for their careful evaluation of our manuscript and for their constructive comments. Below we address the remaining issues raised in the decision letter.

########

Some of the subjective statements go beyond what is supported by the current evidence. Please adjust your claims and arguments, particularly in the “Discussion” and “Conclusion” sections.

########

We revised the “Discussion” and “Conclusion” sections to ensure that interpretive statements remain closely aligned with the observational nature of the data. In particular, we softened several claims regarding visitor behaviour, constructivist learning, and the practical implications of the findings. The corresponding revisions are highlighted throughout the marked-up manuscript.

########

In the second-to-last paragraph of the “Discussion” section, please clarify the theoretical, methodological, or practical limitations of the conclusions or the proposed model.

########

The manuscript already contained a dedicated limitations paragraph at the end of the “Conclusion”. Rather than duplicating these points in the “Discussion”, we expanded the preceding concluding paragraph by explicitly clarifying the theoretical scope of the conclusions while retaining the dedicated limitations section.

Original paragraph:

“Our research draws on a large and diverse dataset, gathering observations of more than 2,000 individuals across five exhibition units and employing a mixed-method protocol combining quantitative and qualitative measures. This design provides a comprehensive perspective on visitor behavior in a constructivist museum environment, offering insights into how exhibit design and family dynamics interact in practice. These findings contribute to ongoing discussions about the translation of constructivist principles into museum settings and may inform strategies for creating exhibitions that balance playfulness with educational depth.”

Revised passage:

“Our research draws on a large and diverse dataset, encompassing observations of more than 2,000 individuals across five exhibition units and employing a mixed-method protocol combining quantitative and qualitative measures. This design provides a comprehensive perspective on visitor behavior in a constructivist museum environment, offering insights into how exhibit design and family dynamics interact in practice. Our findings derive from a single constructivist museum setting and describe observable patterns of visitor behaviour rather than providing a general assessment of constructivist museum learning. Although visitor interaction and engagement represent only indirect indicators of learning processes, they contribute to ongoing discussions about the application of constructivist principles in museum settings and may inform strategies for creating exhibitions that balance playfulness with educational depth. In this respect, the study also contributes to research on the educational role of museums and other cultural institutions consistent with the objectives of SDG 4 (Quality Education).”

The subsequent limitations paragraph (retained from the previous version) continues to address additional methodological limitations, including the limited number of observation points and the absence of individual-level analyses.

########

You may draw a connection to the SDGs at the end of the conclusion.

########

Following the reviewer’s suggestion, we added a concluding sentence placing the study in the broader context of SDG 4 (Quality Education):

“In this respect, the study also contributes to research on the educational role of museums and other cultural institutions, consistent with the objectives of SDG 4 (Quality Education).”

Attachment Submitted filename: Response_to_Reviewers_PONE-D-25-27943R1 (1).pdf

# Editor report: Decision Letter 2

**DOI:** 10.1371/journal.pone.0355543.r005

**Authors:** Weicong Li

23 Jul 2026

Too young to ask their own questions: Learning and visitor dynamics in a constructivist museum environment

PONE-D-25-27943R2

Dear Dr. Jehlicka,

We’re pleased to inform you that your manuscript has been judged scientifically suitable for publication and will be formally accepted for publication once it meets all outstanding technical requirements.

Within one week, you’ll receive an e-mail detailing the required amendments. When these have been addressed, you’ll receive a formal acceptance letter and your manuscript will be scheduled for publication.

An invoice will be generated when your article is formally accepted. Please note, if your institution has a publishing partnership with PLOS and your article meets the relevant criteria, all or part of your publication costs will be covered. Please make sure your user information is up-to-date by logging into Editorial Manager at Editorial Manager® and clicking the ‘Update My Information' link at the top of the page. For questions related to billing, please contact billing support.

If your institution or institutions have a press office, please notify them about your upcoming paper to help maximize its impact. If they’ll be preparing press materials, please inform our press team as soon as possible -- no later than 48 hours after receiving the formal acceptance. Your manuscript will remain under strict press embargo until 2 pm Eastern Time on the date of publication. For more information, please contact onepress@plos.org.

Kind regards,

Weicong Li, P.hD

Academic Editor

PLOS One

Additional Editor Comments (optional):

The manuscript’s citation format is non-standard and should use the [no.] format. The current use of parentheses ( ) overlaps with the numerical data reported in the “Results” section, which can be confusing.

Reviewers' comments:

# Editor report: Acceptance letter

**DOI:** 10.1371/journal.pone.0355543.r006

**Authors:** Weicong Li

PONE-D-25-27943R2

PLOS One

Dear Dr. Jehlicka,

I'm pleased to inform you that your manuscript has been deemed suitable for publication in PLOS One. Congratulations! Your manuscript is now being handed over to our production team.

At this stage, our production department will prepare your paper for publication. This includes ensuring the following:

* All references, tables, and figures are properly cited

* All relevant supporting information is included in the manuscript submission,

* There are no issues that prevent the paper from being properly typeset

You will receive further instructions from the production team, including instructions on how to review your proof when it is ready. Please keep in mind that we are working through a large volume of accepted articles, so please give us a few days to review your paper and let you know the next and final steps.

Lastly, if your institution or institutions have a press office, please let them know about your upcoming paper now to help maximize its impact. If they'll be preparing press materials, please inform our press team within the next 48 hours. Your manuscript will remain under strict press embargo until 2 pm Eastern Time on the date of publication. For more information, please contact onepress@plos.org.

You will receive an invoice from PLOS for your publication fee after your manuscript has reached the completed accept phase. If you receive an email requesting payment before acceptance or for any other service, this may be a phishing scheme. Learn how to identify phishing emails and protect your accounts at https://explore.plos.org/phishing.

If we can help with anything else, please email us at customercare@plos.org.

Thank you for submitting your work to PLOS ONE and supporting open access.

Kind regards,

PLOS ONE Editorial Office Staff

on behalf of

Dr. Weicong Li

Academic Editor

PLOS One
